# Not All Trees Sleep the Same—High Temporal Resolution Terrestrial Laser Scanning Shows Differences in Nocturnal Plant Movement

**DOI:** 10.3389/fpls.2017.01814

**Published:** 2017-10-20

**Authors:** András Zlinszky, Bence Molnár, Anders S. Barfod

**Affiliations:** ^1^Balaton Limnological Institute, Centre for Ecological Research, Hungarian Academy of Sciences, Tihany, Hungary; ^2^Ecoinformatics and Biodiversity Section, Department of Bioscience, Aarhus University, Aarhus, Denmark; ^3^Department of Photogrammetry and Geoinformatics, Budapest University of Technology and Economics, Budapest, Hungary

**Keywords:** circadian plant movement, laser scanning, tree canopy movement, 3-dimensional modeling, tree physiology, turgor, chronobiology, circadian rhythm

## Abstract

Circadian leaf movements are widely known in plants, but nocturnal movement of tree branches were only recently discovered by using terrestrial laser scanning (TLS), a high resolution three-dimensional surveying technique. TLS uses a pulsed laser emitted in a regular scan pattern for rapid measurement of distances to the targets, thus producing three dimensional point cloud models of sub-centimeter resolution and accuracy in a few minutes. Here, we aim to gain an overview of the variability of circadian movement of small trees across different taxonomic groups, growth forms and leaf anatomies. We surveyed a series of 18 full scans over a 12-h night period to measure nocturnal changes in shape simultaneously for an experimental setup of 22 plants representing different species. Resulting point clouds were evaluated by comparing changes in height percentiles of laser scanning points belonging to the canopy. Changes in crown shape were observed for all studied trees, but clearly distinguishable sleep movements are apparently rare. Ambient light conditions were continuously dark between sunset (7:30 p.m.) and sunrise (6:00 a.m.), but most changes in movement direction occurred during this period, thus most of the recorded changes in crown shape were probably not controlled by ambient light. The highest movement amplitudes, for periodic circadian movement around 2 cm were observed for *Aesculus* and *Acer*, compared to non-periodic continuous change in shape of 5 cm for *Gleditschia* and 2 cm for *Fargesia*. In several species we detected 2–4 h cycles of minor crown movement of 0.5–1 cm, which is close to the limit of our measurement accuracy. We present a conceptual framework for interpreting observed changes as a combination of circadian rhythm with a period close to 12 h, short-term oscillation repeated every 2–4 h, aperiodic continuous movement in one direction and measurement noise which we assume to be random. Observed movement patterns are interpreted within this framework, and connections with morphology and taxonomy are proposed. We confirm the existence of overnight “sleep” movement for some trees, but conclude that circadian movement is a variable phenomenon in plants, probably controlled by a complex combination of anatomical, physiological, and morphological factors.

## Introduction

Circadian movement of leaves includes the process of nyctinasty, already known to the ancient Greeks (Bretzl, 1903) but first described by Darwin (Darwin and Darwin, 1880). The leaves of many shrubs and trees actively change position during the night. In some fabaceous trees this phenomenon is particularly striking. In a recent series of experiments by Puttonen et al. (2016) terrestrial laser scanning (TLS) was applied to detect well-defined circadian movement rhythm of the branches and the trunk of the temperate tree *Betula pendula*. TLS (Dassot et al., 2011) allows for detailed measurement of the distance from sensor to a target object by measurement of the travel time of short laser pulses emitted from a given position in a known direction. Since the measurements are conducted during very short time (in the order of milliseconds), a high repetition rate is possible, which together with an equidistant scan pattern allows regular sampling of the target objects. The output of such a measurement is a set of points with positioning coordinates and the amplitude of the reflected signal as an attribute. By employing TLS, Puttonen et al. (2016) created detailed 3-dimensional models of trees at hourly intervals during the night. Their results show that tree branches moved up to 8 cm downwards during the night and returned to their original position by daybreak. By scanning two *Betula* trees nearly 2,000 km from each other, they proved that this phenomenon is probably an attribute which is unique to individual plant species. This seminal discovery opened up many new questions. What is the adaptive advantage of sleep movement (as Darwin called it and as we will also call nocturnal changes in plant shape)? What are the processes governing sleep movements and how are these linked to ambient light, temperature, humidity, and soil water content? Are the movements driven by an internal circadian clock or induced externally by photoperiodism? Are patterns of sleep movement characteristic for certain taxonomic groups?

Our aim is to gain an overview of the variability of plant circadian movement in seed plants. Our method measures movement as the concerted displacement of branches and leaves. Under conditions monitored for light, humidity, temperature, and (lack of) wind, we surveyed a wide taxonomic range of trees and shrubs grown in containers. The experiment was set up to compare sleep movement across taxonomic groups and different types of phenology and morphology. Advancing the methodology of nocturnal tree movement quantification was outside the scope of this mainly qualitative study.

## Materials and methods

### Measurement setup

#### Timing

The study was carried out on 16–17 September 2016, close to the autumn solstice when day and night are equally long. The first laser scanning was conducted at 6:30 p.m. and repeated every 30 min until 8:00 p.m. Hereafter, the scans were repeated every hour until 4:00 a.m. the next day. Finally a series of half-hourly scans were conducted until 7:00 a.m.

#### Location and general environment

The experiment was carried out at a gardening shop at Balatonfüred, Western Hungary (N46°56′16.91′′E17°51′42.48′′), which has developed an open-air nursery for small-sized container grown woody plants. The shrubs and young trees were kept for at least half a year in the nursery, where they were watered on a daily basis. All the plants included in this study have thus been exposed to exactly the same weather and water conditions for at least 6 months. To avoid the expected effect of wind action we conducted the experiment in a greenhouse under construction, which had a glass roof, but no walls. During the full duration of the experiment weather conditions were stable with no wind and no precipitation or condensation. The nearest streetlights were more than 1 km away. During the overnight experiment we made great effort to exclude all outdoor lights and restrict the use of torch light to a minimum. A full moon allowed us to operate the scanner and read the instruments without an additional light source.

#### Experiment design and studied plants

The choice of plants was somewhat restricted to what was available to us at site. Nevertheless, we were able to include in the experiment a broad range of growth forms (trees, shrubs, bamboos, and palms), leaf phenologies (evergreen and deciduous), leaf morphologies (entire, dissected, needle-shaped, and scaly), taxonomic groups, and ecoclimatic ranges (tropical, subtropical, temperate). Although high priority was given to include tall and dense plants, the height was restricted by the size of the containers. For most species we were unable to include more than one individual.

**Table:** 

**Vernacular name**	***Scientific name***	***Height of studied specimen [m]***	***Crown diameter of studied specimen [m]***	**Taxonomy (Chase et al., 2016)**	**Origin**	**Phytoclimatic region**	**Phenology**
Nootka cypress	*Chamaecyparis nootkatensis* (D.Don) Spach	*2.75*	*2.2*	Gymnosperm	NE US & Pacific Canada	Boreal	Evergreen
Japanese larch	*Larix kaempferi* (Lamb.) Carr.	*2.8*	*0.9*	Gymnosperm	SE Asia	Subalpine	Deciduous
Warty birch	*Betula pendula* Roth.	*3.72*	*1*	Fabid	Europe & SW Asia	Nemoral to boreal	Deciduous
Himalayan birch	*Betula utilis* D. Don	*1.35*	*1.1*	Fabid	Himalayas	Alpine	Deciduous
Southern magnolia	*Magnolia grandiflora* L.	*2.75*	*1.4*	Basal angiosp.	SE US	Subtropical	Evergreen
Austrian pine	*Pinus nigra* Arnold	*1.9, 3.7 (two trees)*	*1.1, 1.9 (two trees)*	Gymnosperm	Europe	Subalpine	Evergreen
Cedar of Libanon	*Cedrus libani* A. Rich	*2.75*	*1.1*	Gymnosperm	E. Mediterran.	Subalpine	Evergreen
Japanese maple	*Acer palmatum* Thunb.	*3.8*	*2*	Malvid	NE Asia	Temperate	Deciduous
Horse chestnut	*Aesculus hippocastanum*L.	*3.75*	*1.1*	Malvid	SE Europe	Temperate	Deciduous
White mulberry	*Morus alba* L.	*2.75*	*1.5*	Fabid	N China	Subtropical to temperate	Deciduous
Pedunculate oak	*Quercus robur* L.	*3.25*	*1.3*	Fabid	Europe	Nemoral	Deciduous
Chokecherry	*Prunus virginiana* L.	*3.2*	*0.9*	Fabid	N America	Temperate	Semi-evergreen
Olive	*Olea europea* L	*2.6*	*2.3*	Lamid	E. Mediterran.	Subtropical	evergreen
Prage viburnum	*Viburnum x pragense*	*1.5*	*1.5*	Campanulid	n.a.	n.a.	semi- evergreen
Persian silk tree	*Albizia julibrissin* Durazz.	*3.5*	*1.8*	Fabid	SW & E Asia	Subtropical	deciduous
Honey locust	*Gleditsia triacanthos L*.	*3.5*	*1.7*	Fabid	N America	Subtropical to temperate	deciduous
Oleander	*Nerium oleander L*.	*1.75*	*1.95*	Lamid	SW Asia	Subtropical	evergreen
Pagoda tree	*Styphnolobium japonicum* (L.) Schott (syn. *Sophora japonica* L.)	*1.8*	*0.7*	Fabid	China	Subtropical to temperate	deciduous to evergreen
Red tip photinia	*Photinia x fraseri*	*2.7*	*1.4*	Fabid	n.a.	n.a.	semi- evergreen
Umbrella bamboo	*Fargesia murielae* T.P.Yi.	*2.65*	*1*	Monocot	China	Temperate to subtropical	evergreem
European fan palm	*Trachycarpus fortunei* (Hook)	*2.3*	*2*	Monocot	W. Mediterran.	Subtropical	evergreen

### Measurement techniques

#### Ambient sensors and instruments

Ambient air temperature and humidity were recorded by placing six climate loggers on the trunks of six selected trees and near the LIDAR sensor (Easylog USB™, Lascar Electronics®, UK), and temperature loggers (HOBO Pendant™) in the soil containers of the same trees. These were set to collect data every 10 s. Wind speed was measured before each laser scanning measurement cycle using a simple hand-held ball anemometer (Dwyer Instruments, Michigan City, IND, US). At the same time ambient light was recorded using a LabSphere™ integrating sphere mounted on a Li-Cor™ data logger, within the greenhouse frame as well as outside.

#### Terrestrial laser scanning instrument and measurement parameters

Laser scanning can produce a high-density point cloud that allows for dynamic 3D modeling of complex shapes in few minutes. For 3D data acquisition, a phase-based Terrestrial Laser Scanner (TLS) was applied. FARO™ Focus S120 (FARO Technologies, Lake Mary, FL, US) operates a Class 3 laser with 905 nm wavelength. Acquisition parameters were set to capture 7 × 10^9^ points in 15 min with highest quality. Based on instruments data sheet (FARO, 2013), the distance measurement has 0.95 mm accuracy on a solid, well-defined target while angular deviation has ± 2 mm effect on accuracy at 25 m object distance (Note: in our case the furthest tree had an object distance of 15 m). Each raw scan requires 3 GB storage space, however, only ~10% of the points are representing trees. Therefore, the first step of post-processing was to reduce the amount of data manually based on object distance and direction, removing all points that were outside the greenhouse frame. Reducing data amount allowed efficient data handling and comparison. The second step was to co-register the time series of the measurements. All measurements were captured from the same scanner position; however, the scanner battery and storage device (SD card) had to be replaced during the measurement session. Because this could potentially cause minimal changes in the scanner position relative to the object, we choose target based registration with reference spheres, but also including the steel girder frame of the greenhouse as an additional source of reference surfaces. Altogether 18 scans were processed; maximum mean tension of registration between the reference (first) scan and any other scans is 2.3 mm. The first scan was selected as a fixed position, but global registration was applied for network adjustment. Assuming that all the scans were captured from the same position, no error propagation was expected from the network.

### Data analysis and visualization

After removal of terrain and roof points we viewed the point clouds belonging to individual plants in 3D using FugroViewer® and in 2-dimensional height models which were obtained by rasterizing the maximum height in each cell in 10 cm intervals. The 2D outlines were digitized in GIS with a margin of at least 10 cm, and further refined with the point cloud viewer to exclude all points belonging to the greenhouse structures and the plant container. Wherever the canopy started more than ~1 m above the soil level of the container, we also excluded the lower part of the trunk from subsequent analysis via a minimum height limit in order not to misinterpret canopy height changes. For each plant, this frame defined by the horizontal and the vertical limits of the canopy was kept constant for individual exports of the point clouds acquired during the scannings. No further editing of the raw data was undertaken. We used the procedure described by Puttonen et al. (2016) to subdivide individual point clouds into height percentiles that were exported to Excel. The height of each percentile during the first survey (6:30 p.m.) was used as baseline, and the changes of percentile heights with respect to this baseline were plotted during the course of the experiment. All changes in percentile height exceeding 0.5 cm were interpreted as movement. Percentiles are height thresholds that separate the point cloud horizontally into sets of equal counts of points amounting always to 10% of the total point count for each survey. Where the branches and/or leaves of a plant are displaced, this is followed by the height distribution of the point cloud and thus also the height percentiles. However, depending on the crown shape, the effect of canopy displacement on the changes in height percentiles may vary considerably. For trees with a uniform (near-cylindrical) crown shape extending from the ground right upwards, the interpretation of percentile height changes is straightforward. For trees with other shapes, the narrower parts such as the trunk or crown apex have the same number of points between percentiles, but spread across a wider height interval. Height changes in any part of the canopy affect all percentiles, and such narrow parts amplify these effects, creating artifacts of high displacement of these percentiles. To keep track of such effects, we indicated in gray the curves pertaining to the percentiles of the narrow tree parts. To render figures comparable we excluded data from percentiles pertaining to the most narrow parts of the tree, where the extent across was less than 50% of the maximum crown diameter. The movement for the percentiles that had more than 50% of the maximum crown diameter was also averaged to create a mean displacement value for each studied point in time. The extent and nature of canopy movement was visually interpreted from these graphs, checking time distances between local and general minimum and maximum points and the vertical extent of displacement, without further numeric data analysis, in accordance with Puttonen et al. (2016).

## Results

### Ambient conditions

Wind speed was measured to be 0 m/s during all measurements. Ambient light level inside the greenhouse was 63 mmol/s/m^2^ at 6:30 p.m., 9.3 at 7:00 p.m., and dropped to 0.05 at 7:30 p.m., remaining at or below this value until 6:30 a.m., when 2.4 mmol/s/m^2^ was measured, increasing to 33 mmol/s/m^2^ by the last measurement at 7:00 a.m. Air temperature decreased gradually from 24°C at 6:30 a.m. to 20°C at midnight and further to 17°C around 3:00 a.m., where it slowly increased to 18°C by 7:00 a.m. All air temperature sensors recorded the same temperature within ± 1°C. Relative humidity was around 65% for all sensors placed on tree trunks (and 55% for the sensor near the LIDAR), and increased continuously throughout the studied timeframe with small fluctuations of 1–2% that were closely similar for all trees, up to 95% at 7:00 a.m.

### Observations of tree position changes

All the plants measured showed movement of at least part of the crown that exceeded our noise threshold defined here as 0.5 cm. Most trees showed periodic movement. Either they revealed true sleep movement by returning to the early evening position by next morning, or they showed shorter term periodic movement, or both. Additionally, some trees showed unidirectional movement and did not return to their starting position within the measurement timeframe.

The most striking result obtained in this study was the large variation revealed in nocturnal tree movement patterns across the seed plants. *Betula utilis* (Figure S7) and *Olea europea* (**Figure 4**) displayed the least movement, where the mean of all crown height percentiles never exceeded the preset threshold of 0.5 cm for noise. The legume tree *Gleditschia triacanthos* (**Figure 14**) represented the opposite extreme by moving part of its crown up to 9 cm during the scannings. Practically all of the remaining trees showed oscillations in movement and shape of the crown of 0.5–1 cm with cycles that were shorter than the observed timeframe, typically a few hours.

**Figure 1:**
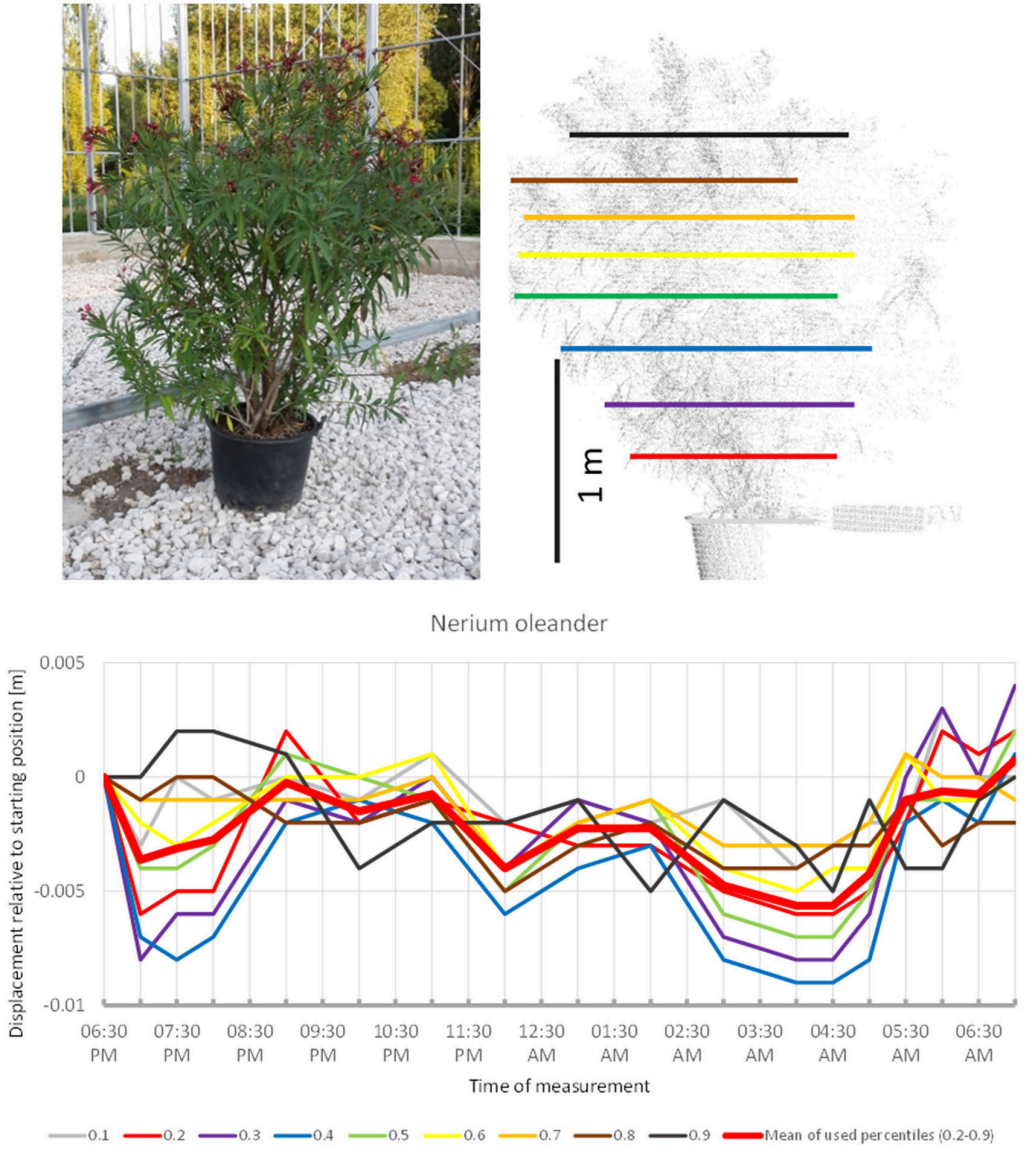
Point cloud height percentiles and their displacement (m) in time relative to the starting position, for *Nerium oleander*. Percentiles not used for calculating mean movement are shown in gray. Note that movement pattern resembles typical sleep motion with some oscillation superimposed.

*Nerium oleander* (Figure 1) revealed a pattern reminiscent of the overnight movement recorded in uncultivated *B. pendula* trees by Puttonen et al. (2016). Our scanning captured a slight downwards movement between 6:30 p.m. and 7:00 p.m. followed by a phase of upwards movement peaking at 9:30 p.m. Hereafter, the crown moved downwards with minor oscillation until 4:30 a.m. The remaining part of the morning the crown moved upward to resume the position recorded at the start of the experiment. Although only minor movements below 1 cm were captured during the scanning, the recorded patterns were very consistent across the crown height percentiles. It should be noted that the overall crown shape of this shrub was relatively even with no pronounced narrow parts.

**Figure 2:**
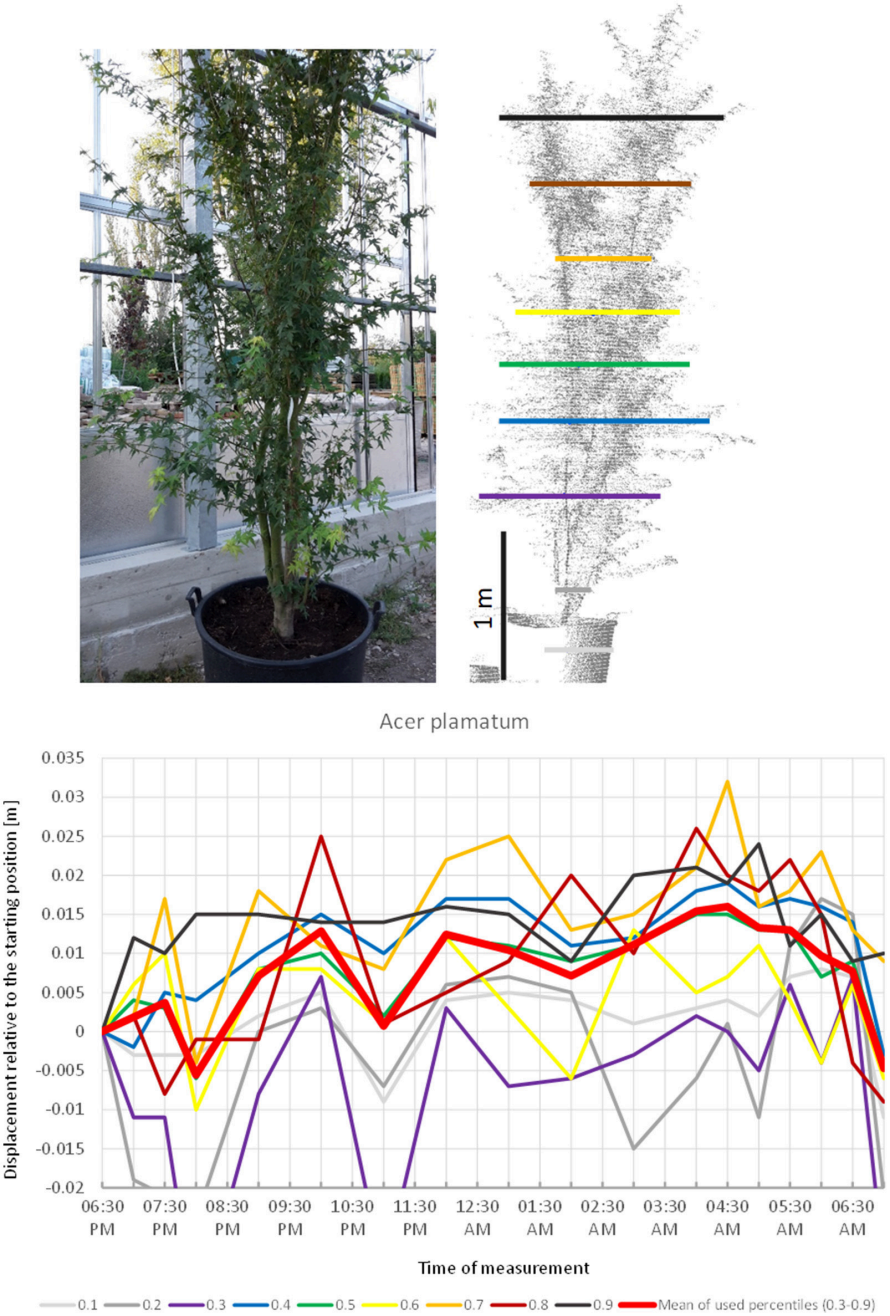
Point cloud height percentiles and their displacement (m) in time relative to the starting position, for *A. palmatum*. Percentiles not used for calculating mean movement are shown in gray. Circadian movement starts upwards and returns close to starting position.

*Acer palmatum* (Figure 2) showed a pronounced circadian cycle as well, but in the exact opposite direction: crown height percentiles were observed to move upwards reaching the highest point (+1.5 cm) at 4:30 a.m. Hereafter, the percentiles gradually moved downwards until next morning when the canopy had almost resumed the starting position.

**Figure 3:**
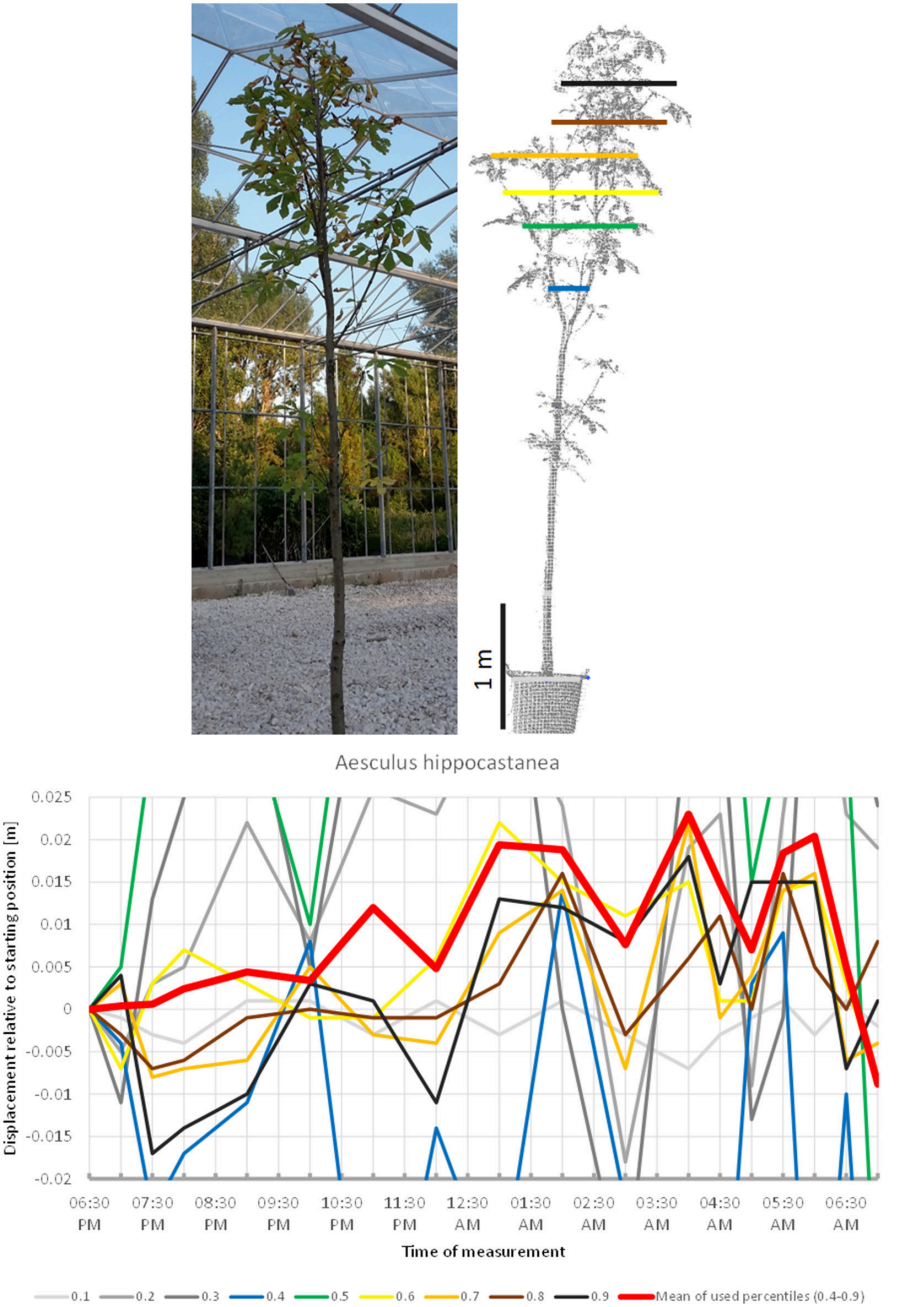
Point cloud height percentiles and their displacement (m) in time relative to the starting position, for *Aesculus hippocastanea*. Percentiles not used for calculating mean movement are shown in gray. Circadian movement is upwards, with intensive oscillation superimposed.

The scanning of *Aesculus hippocastanea* (Figure 3) revealed a similar pattern of upward movement in the evening peaking at 04:00 a.m. and gradual return to the start position at 07:00 a.m. However, this overall pattern was interfered by rapid changes in height, which caused the mean of the crown height percentiles to shift more than 1 cm downwards between 04:00 a.m. and 05:00 a.m.

**Figure 4:**
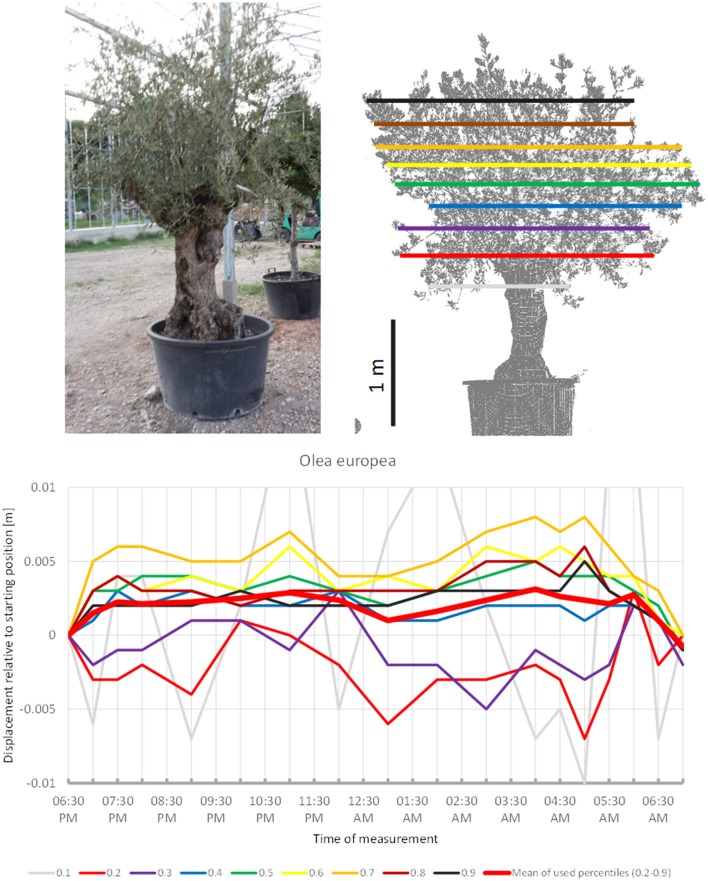
Point cloud height percentiles and their displacement (m) in time relative to the starting position, for *Olea european*. Percentiles not used for calculating mean movement are shown in gray. Slight circadian movement starts upwards and returns to starting position, oscillation of mean is not prominent.

Although sleep movements were detected in the individual crown height percentiles of *O. europea* (Figure 4) the displacement of the mean of all percentiles never exceeded our preset detection threshold of 0.5 cm. The lower branches moved downwards before 07:00 p.m., upwards between 09:00 p.m. and 11:00 p.m., downwards between 01:00 a.m. and 05:30 a.m. and upwards again hereafter. The upper branches moved in quite the opposite pattern, reaching the highest point at 05:00 a.m., and resuming the starting position in the morning. The mean of the percentiles revealed an overall upward trend in the early evening, a more or less constant height during the night and returned to the start by morning; however, it should be noted that the amplitude was only 0.3 cm.

**Figure 5:**
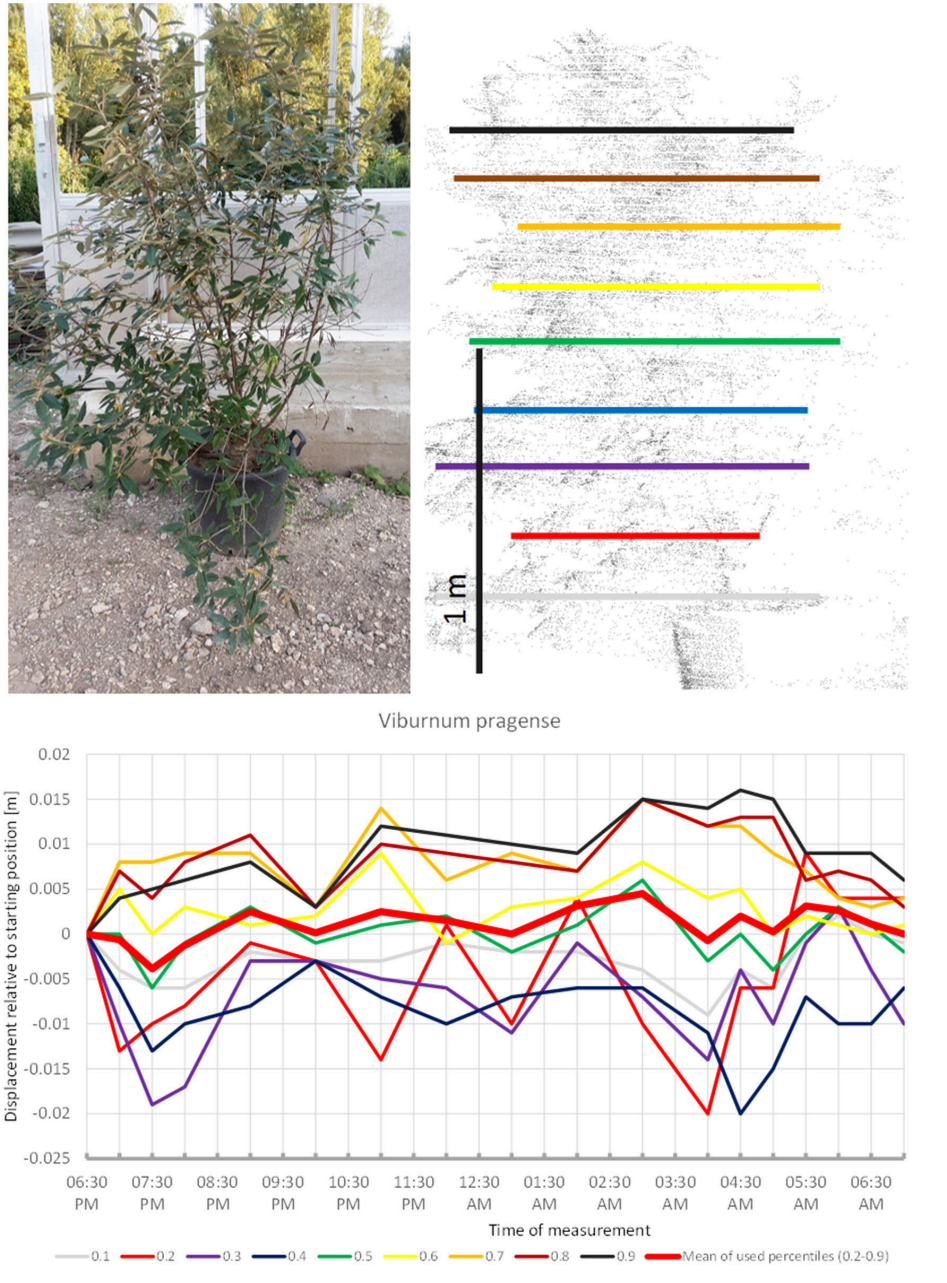
Point cloud height percentiles and their displacement (m) in time relative to the starting position, for *Viburnum pragense*. Percentiles not used for calculating mean movement are shown in gray. Note that lower percentiles move downwards while higher ones move up.

*Viburnum pragense* (Figure 5) moved similarly but with an even longer upward phase and perhaps some short-term periodicity superimposed. The lower branches moved downward whereas the upper branches seemed to move up almost continuously until around 4:30, when the movement was reversed and both the lower and the upper branches of the canopy returned close to their starting position. This results in the displacement amplitude of the mean of all used percentiles barely reaching 0.5 cm.

**Figure 6:**
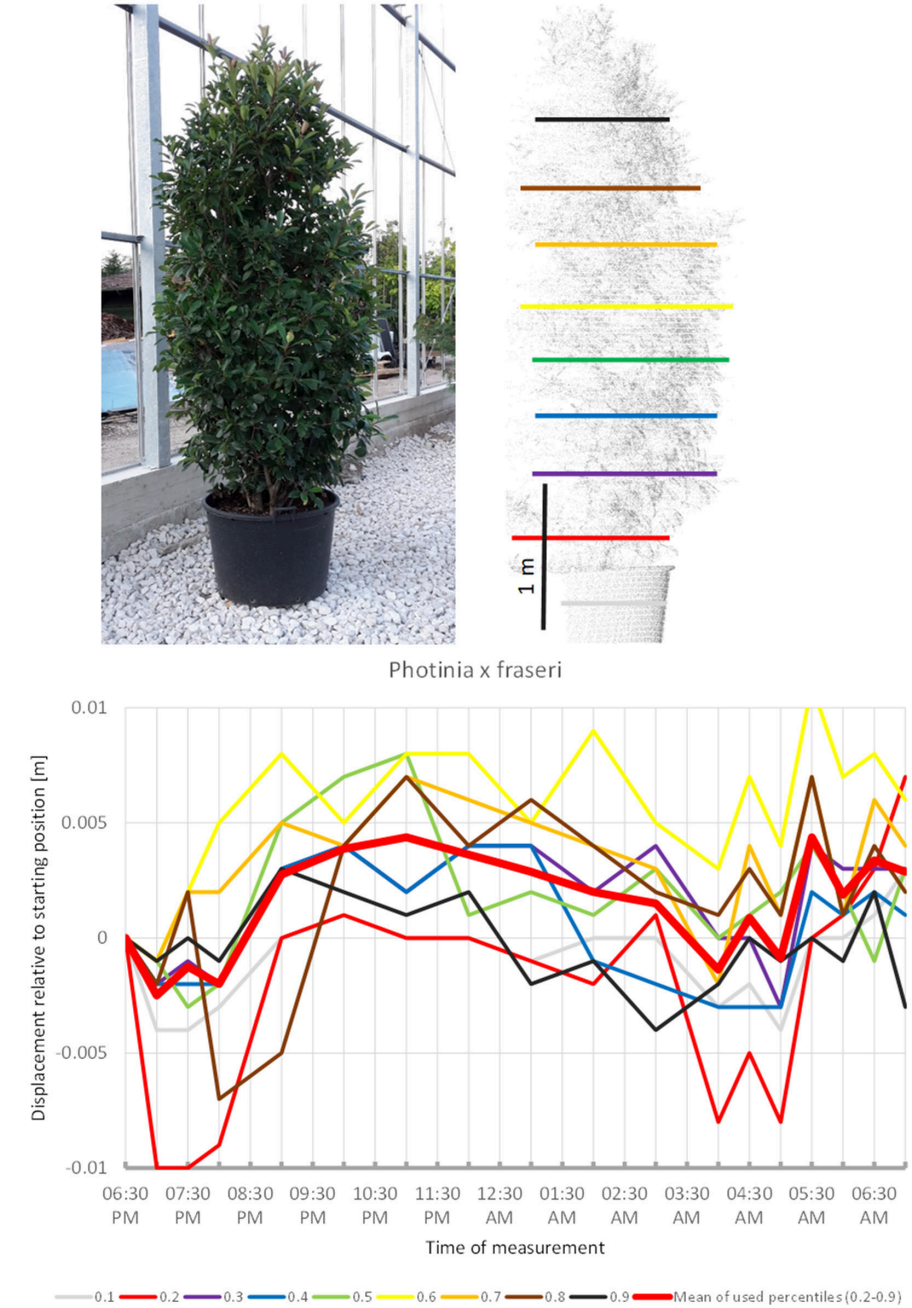
Point cloud height percentiles and their displacement (m) in time relative to the starting position, for *Photinia x fraseri*. Percentiles not used for calculating mean movement are shown in gray. This plant seems to have an overnight phase shorter than 12 h.

*Photinia x fraseri* (Figure 6) also showed movements similar to the upward sleep pattern detected in the species above: most crown height percentiles increased in height until 11:00 p.m. (compared to 10:00 p.m. recorded for most other plants), then slowly moved downwards until a minimum was reached between 4:00 and 5:00. However, prior to 8:00 p.m. and after 04:00 a.m., we recorded much more variation in point heights, which suggests that this shrub might have a shorter period of only about 8 h of sleep movement.

**Figure 7:**
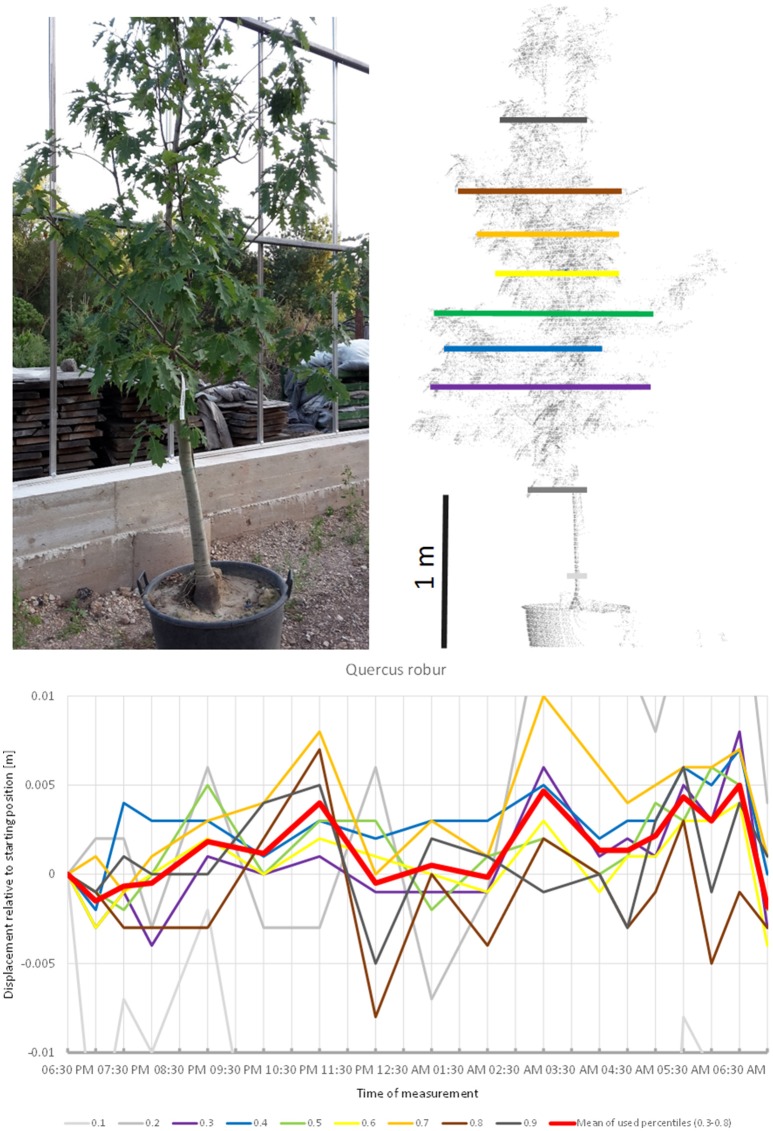
Point cloud height percentiles and their displacement (m) in time relative to the starting position, for *Quercus robur*. Percentiles not used for calculating mean movement are shown in gray. This movement pattern can be interpreted as two cycles, always returning to the mean position.

*Quercus robur* (Figure 7) seemed to carry out two cycles of movement. The first one started at 07:00 p.m., peaked at 11:00 p.m., and returned to the starting position around midnight. The second cycle started at midnight, peaked at 5:00 a.m. and resumed the starting position around 7:00 a.m.

**Figure 8:**
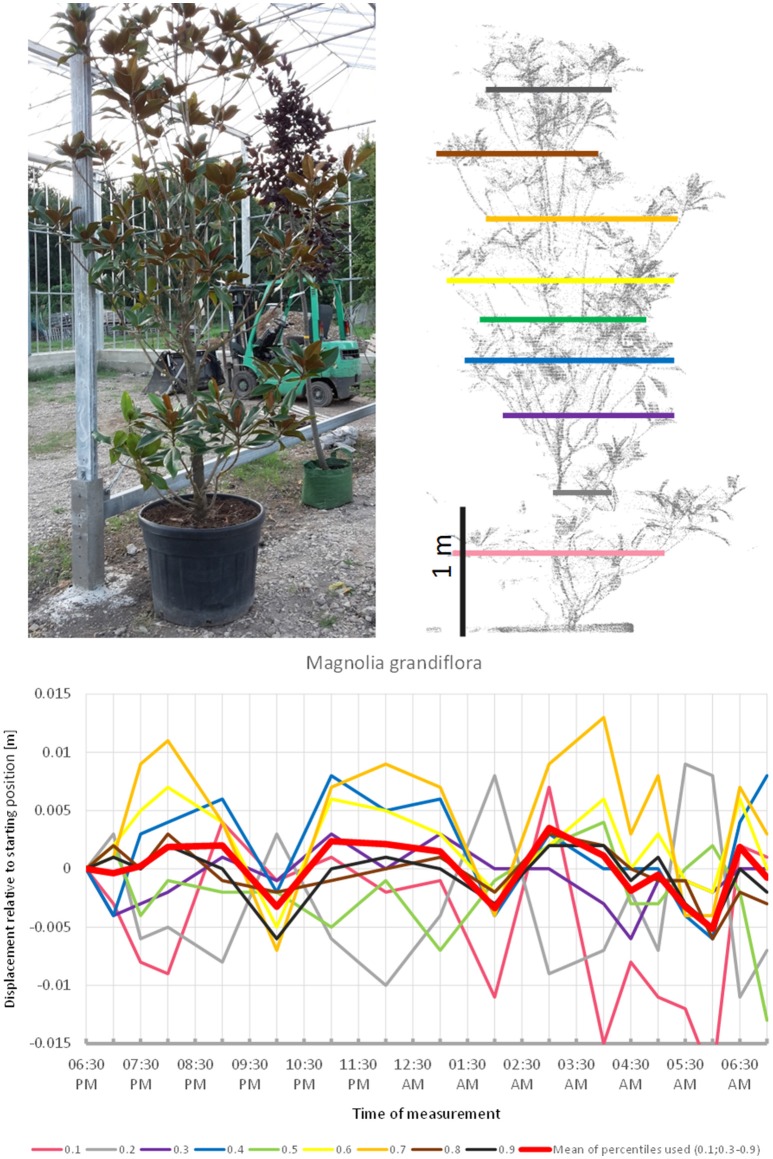
Point cloud height percentiles and their displacement (m) in time relative to the starting position, for *Magnolia grandiflora*. Percentiles not used for calculating mean movement are shown in gray. Movement pattern implies domination of short-term oscillation, with three cycles during the measurement period.

*Magnolia grandiflora* (Figure 8) apparently did not follow a diurnal cycle but moved in cycles of ~3 h, peaking at 8:00 p.m., midnight, and 4:00 a.m. respectively. This pattern was quite distinct from the other species included in the experiment. The fact that the start and end positions of the branches were similar, and that the movement of the individual height percentiles was more or less synchronized strongly suggests that this recorded pattern was not just an artifact of noise. The maximum displacement of the mean of all percentiles is exactly −0.5 cm, which equals to our noise threshold. However, this is partly an effect of the lowest percentile, which moved in the opposite direction relative to the rest of the tree, thus resulting in lower average movements.

**Figure 9:**
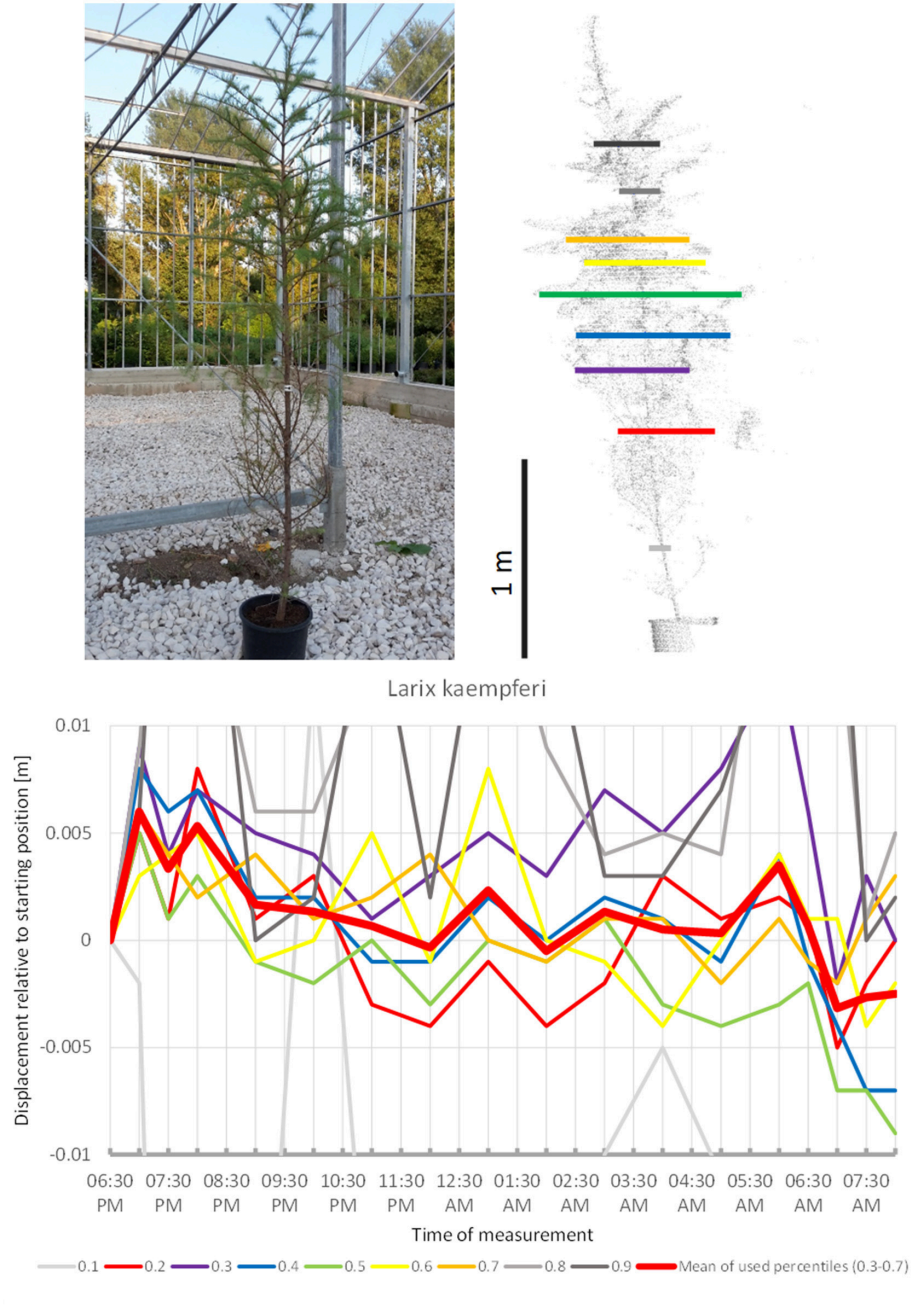
Point cloud height percentiles and their displacement (m) in time relative to the starting position, for *Larix kaempferi*. Percentiles not used for calculating mean movement are shown in gray.

Among the Gymnosperms, *Larix kaempferi* (Figure 9) showed upward movement during the first hours of the survey and then continued downward for the rest of the night, with two short peaks at 01:00 a.m. and 06:00 a.m. It returned relatively close to its start position by the end of the experiment in the morning. Nevertheless, the recorded movement pattern seemed to deviate from the sleep patterns of the trees above.

**Figure 10:**
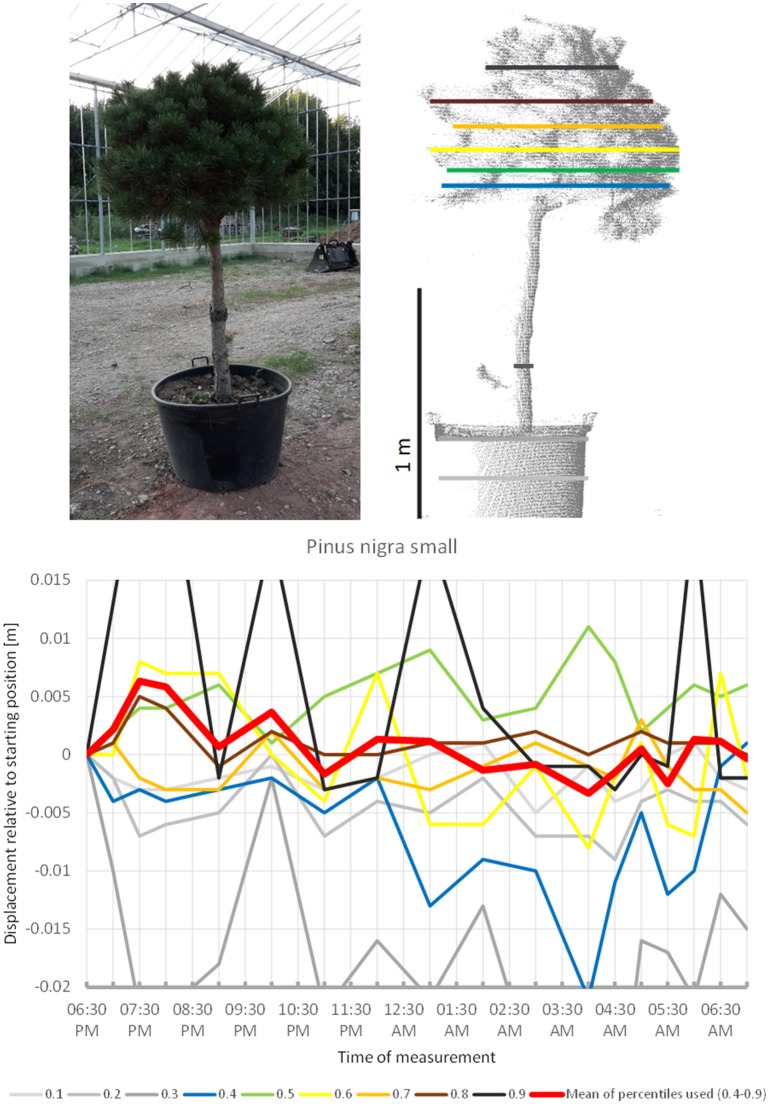
Point cloud height percentiles and their displacement (m) in time relative to the starting position, for the smaller *Pinus nigra* tree. Percentiles not used for calculating mean movement are shown in gray. Fluctuation around a mean returns to the original position by the end of the survey in the morning.

The smaller of the two *Pinus nigra* individuals included in the experiment, *P. nigra* 2 (Figure 10), moved upwards until 7:30 p.m., downward until 04:00 a.m., and then returned back to the original position, with some oscillation superimposed. More scannings are needed to clarify whether this pattern form part of a sleep movement or rather represents random oscillations around a mean.

The larger *P. nigra* individual, *P. nigra* 1 (Figure S1), showed extreme fluctuations in the beginning of the scanning. We suspect this to be an artifact of mechanical disturbance at the beginning of the experiment and therefore not go into further detail. Compared to the other species included in the experiment the percentiles are highly variable in between scannings. This may be explained by the large size and long branches of the selected individual. The mean of the percentiles revealed that the tree kept a relatively constant height during most of the experiment, with two peaks at 8:00 p.m. and 05:00 a.m. respectively. It should be noted that this particular tree was almost twice as tall as the other trees included in the study and therefore had to be placed outside the greenhouse. For this reason great caution should be taken when making comparisons with the other species.

*Cedrus libanica* (Figure S2) fluctuated around a mean as well, with peaks around 07:00 p.m. and 3:00 a.m. It stood out, however, by not returning to its original position at the end of the measurement; in fact, the largest displacement from the starting position was observed at the last scan in the morning.

**Figure 11:**
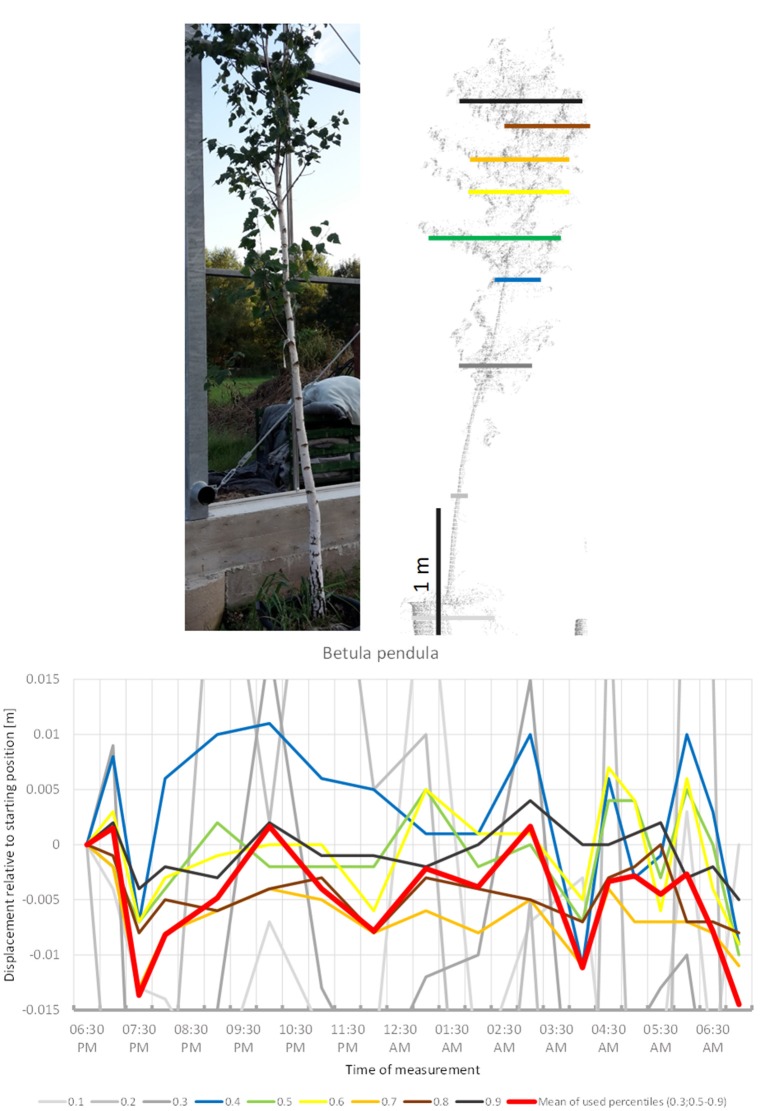
Point cloud height percentiles and their displacement (m) in time relative to the starting position, for *Betula pendula*. Percentiles not used for calculating mean movement are shown in gray. Movement of the tree seems to be dominated by short-term periodicity and does not return to the original position.

*B. pendula* (Figure 11) showed some abrupt downward movements compared to a seemingly constant baseline. Low points are occurred at 8:00 p.m., 04:00 a.m., and 07:00 a.m. This did not resemble classical downward sleep movement. Movement of most crown height percentiles was congruent. The tree did not return to its original position by 07:00 a.m.

**Figure 12:**
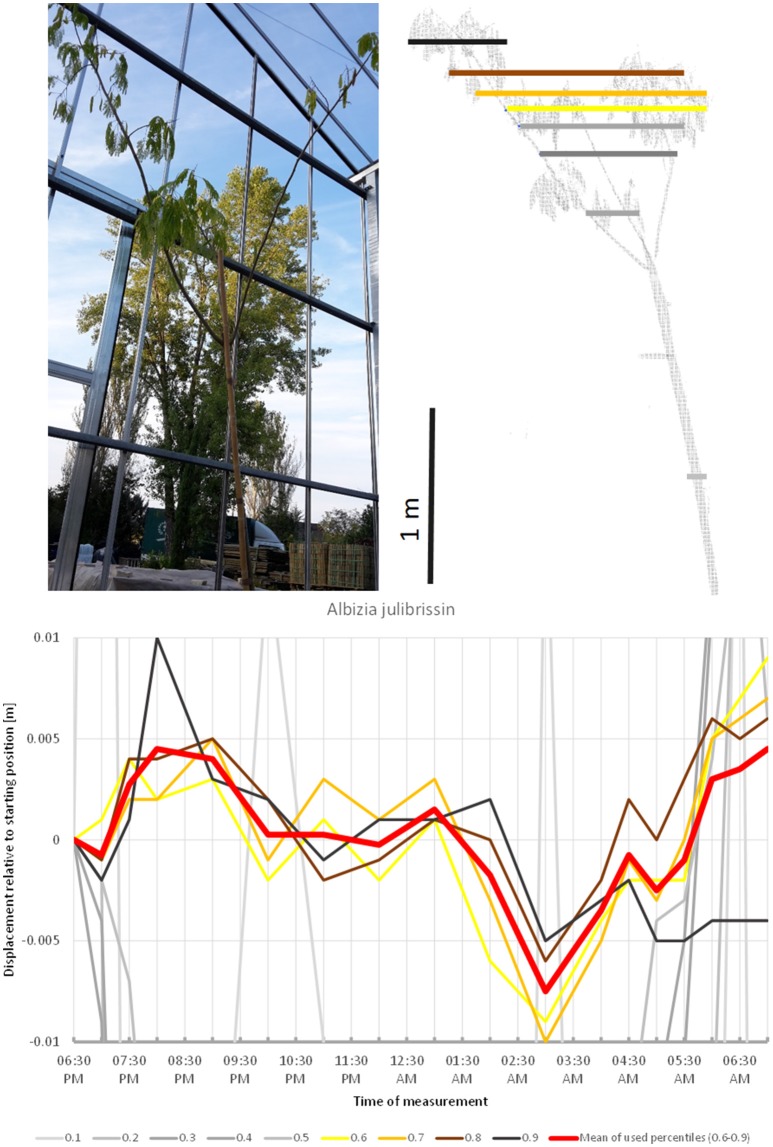
Point cloud height percentiles and their displacement (m) in time relative to the starting position, for *Albizia julibrissin*. Percentiles not used for calculating mean movement are shown in gray. Movement of this tree might be interpreted as a circadian cycle returning to the original position before the end of the measurement.

Many plants belonging to the family Fabaceae with pinnately or double pinnately compound leaves display striking sleep movement of their leaves (nyctinasty). Among the trees included in our experiment *Albizia julibrissin* (Figure 12) showed the most conspicuous movement pattern, even though the leaves entered the sleep position before the scanning was initiated. A substantial part of this movement may be explained by the concerted action of the pulvini of consecutive orders. The scanning revealed upward movement of the crown until ca. 9:00 p.m., followed by downward movement until it reached the lowest point at 03:00 a.m. Hereafter, the crown gradually ascended until the morning. The leaves were observed to have reached their daylight configuration around 6:30 a.m., however, the tree reached its 06:30 p.m. start position already 1 h earlier (5:30 a.m.), and continued to move upward.

**Figure 13:**
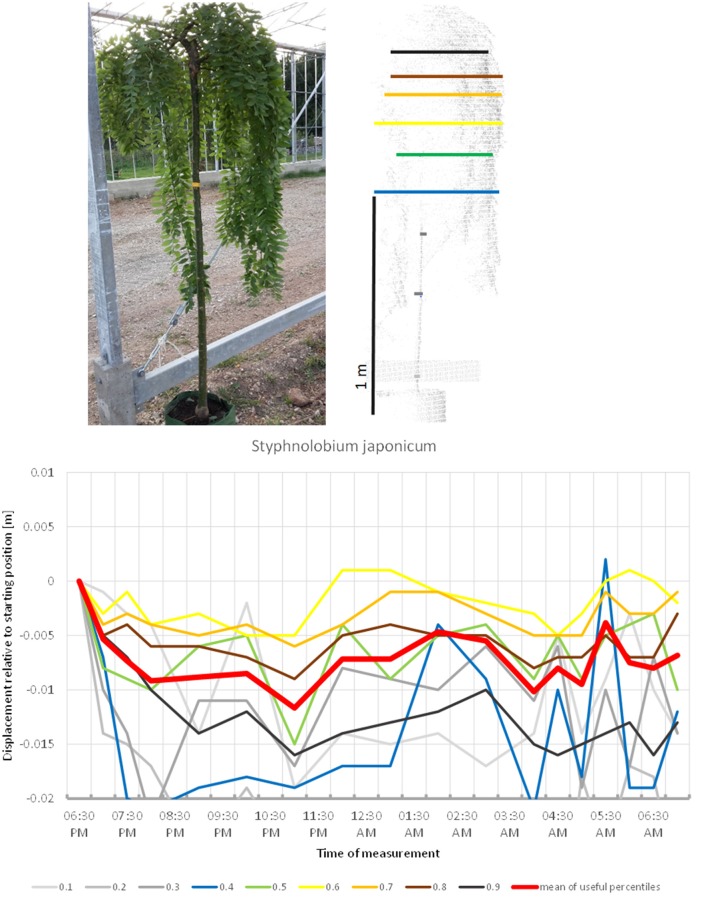
Point cloud height percentiles and their displacement (m) in time relative to the starting position, for *Styphnolobium japonicum*. Percentiles not used for calculating mean movement are shown in gray. It is not clear whether the branch positions would have returned to the start later during the day.

Another fabaceous tree included in the study, *Styphnolobium japonicum*, (Figure 13) stood out by having a pendulous crown, with an overall more or less cylindrical shape. The circadian movement of the leaves contributed only moderately to the overall crown movement in this species. The lower percentiles of the tree revealed most movement, but the broader lower part of the canopy top also moved more than 2 cm down. The crown movement had two low points, one at midnight and one between 4:00 and 5:00. It was not clear whether the night phase of the circadian cycle of this tree was longer than the duration of our experiment and the crown would return to its start position later, or whether the movement was not circadian.

*Prunus virginica* (Figure S3) showed a similar pattern, but the crown moved in the opposite, upward direction apparently right from the beginning of the experiment, with the highest point reached around 4:00 by most of the crown height percentiles. A return to the starting position was, however, not observed for the mean and some percentiles and it remains uncertain whether the branches would have resumed the starting position later during the day or whether the movement was induced by other factors than circadian rhythm.

*Trachycarpus fortunei* (Figure S4) stands out in its growth form by having an unbranched stem. Most crown movement must therefore be attributed to the leaves. This species was characterized by a short downward movement, followed by upward movement of most crown height percentiles until 01:00 a.m., then downward movement until 4:30 and finally upward movement again. It is not clear whether this pattern reflects a short circadian cycle similar to *P. x fraseri*, or whether it is the result of a longer cycle with a return to the starting position later during the day.

**Figure 14:**
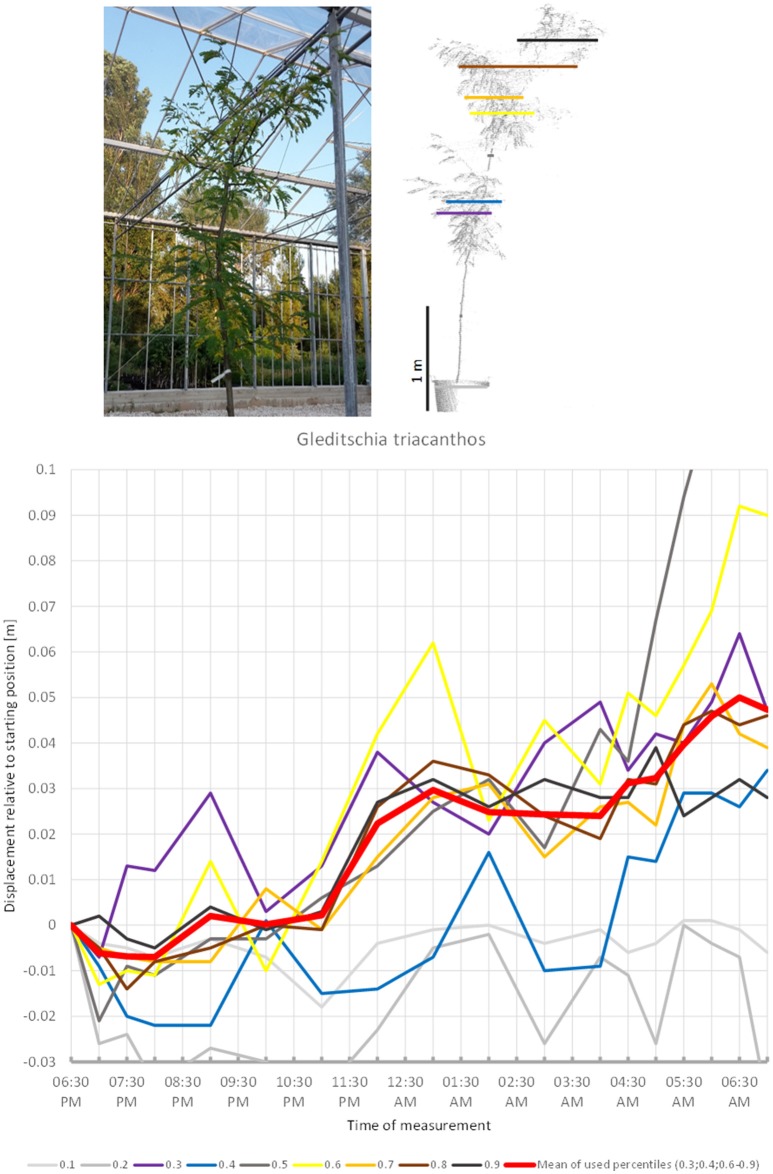
Point cloud height percentiles and their displacement (m) in time relative to the starting position, for *Gleditschia triacanthos*. Percentiles not used for calculating mean movement are shown in gray. Movement of this tree seems to be dominated by aperiodic displacement.

Some trees showed more or less unidirectional movement through the duration of the experiment. In such cases the observed patterns were not sufficiently clear to conclude whether the movements represented sleep or not. The leaves of the third fabaceous tree included in the experiment, *G. triacanthos* (Figure 14) were observed to have entered sleep configuration before the beginning of the experiment. The crown height percentiles of the canopy moved slightly downwards between 06:30 p.m. and 07:00 p.m., then moved upwards by 9:00 p.m. until 01:00 a.m., when a slight downward movement was detected, after which the percentiles showed a clear increase in height again. The last position scanned was situated 5 cm away from the starting position and we did not observe any signs that this would be resumed later.

The crown of *Morus alba* (Figure S5) moved constantly upward during the experiment, with only a slight downward movement around midnight. The duration of the experiment was not sufficiently long to determine whether this pattern was due to sleep movement initiated before the scanning started at 06:30 p.m. or some other processes. However, since this tree appeared to be generally unhealthy, the observed movements might have been determined by other factors than sleep movement.

The bamboo, *Fargesia murielae* is characterized by having multiple stems, each with a uniform modular structure and slender first order branches carrying the leaves (Figure S6). For the lower two crown height percentiles (0.5 and 0.6) we recorded a pattern similar to other plants with sleep motion: a short downward movement between 06:30 a.m. p.m. and 07:00 a.m. p.m., followed by upward movement until 9:00 p.m., down until 04:30 a.m. and back up, but with the original position reached at 5:30 and downwards movement afterwards. This species was excluded from further analysis since the uppermost part of the crown was partly in contact with and supported by the greenhouse frame.

**Figure 15:**
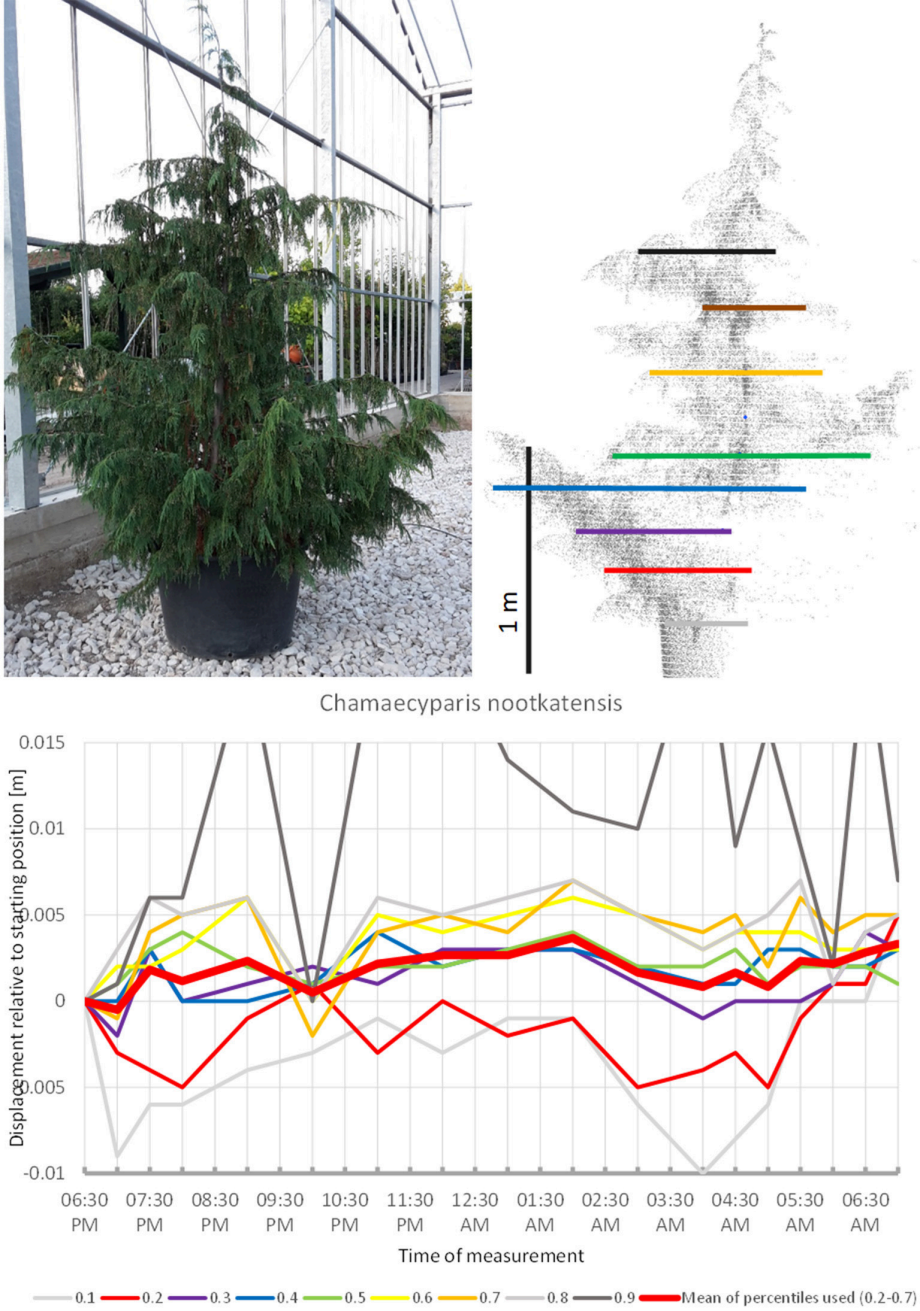
Point cloud height percentiles and their displacement (m) in time relative to the starting position, for *Chamaecyparis nootkatensis*. Percentiles not used for calculating mean movement are shown in gray.

In some of the tress studied, the movement observed can be interpreted as non-cyclic movement superimposed on an overall circadian cycle. This particularly applies to *Chamaecyparis nootkatensis* (Figure 15), which moved upwards during most of the night. At 10:00 p.m. the apical part of the canopy showed an abrupt downward shift (the crown height percentiles 0.6, 0.7, 0.8, and 0.9). An hour later at 11:00 p.m., it had returned to the pre 10:00 p.m. position. The tree reached the highest position at 02:00 a.m. and moved downwards until 5:00 a.m. Hereafter, it moved upwards again but did not resume the staring position. It should be emphasized that this particular tree has small scaly leaves and that the patterns observed were solely a result of branch movements.

Similarly, the crown of *B. utilis* (Figure S7) moved upward as expected until 8:00 p.m. The lowest point was reached at 02:00 a.m., and the highest point at 06:00 a.m. with slight downward movement afterwards. The crown did not return to its original position.

### Summary of results

Changes in crown shape were observed for all studied trees, but clear and well-measurable sleep movements are apparently rare. Ambient light conditions were continuously dark between 7:30 p.m. and 6:00 a.m., but most changes in movement direction occurred during this period, suggesting that they were not controlled by ambient light. *Aesculus* and *Acer* had the highest periodic circadian movement amplitudes of the crown amounting to 2 cm, whereas the highest aperiodic change in crown shape was observed for *Gleditschia* (5 cm) and *Fargesia* (2 cm). We detected some shorter-period oscillation with cycles of 2–3 h and amplitudes between 1 and 0.5 cm, close to the margin of our measurement accuracy. Movement patterns recorded can be attributed to in three major types: circadian movement in an 8–12 h cycle (*Nerium, Acer, Aesculus, Viburnum, Photinia*), periodic movement between 6 and 2 h (*Quercus, Magnolia, Pinus, Cedrus*), and non-periodic, unidirectional movements (*Styphnolobium, Prunus, Gleditschia, Morus*). The remaining trees studied (*Olea, Betula, Larix, Trachycarpus, Fargesia, Chamaecyparis*) either show a combination of these basic movement forms or low-amplitude movements close to the margin of our accuracy or an apparently random pattern.

## Discussion

### Measurement accuracies and their effects on the results

The scanning method used here has a nominal ranging accuracy of 0.003 m on solid, fixed targets. This signifies that theoretically the results presented here can be influenced by errors in position measurement. However, since these effects are probably random, their influence can be reduced by ensuring a large number of points in each height percentile. The differences in the number of points acquired for each tree during the individual scanning sessions fluctuate slightly (± 1%) mainly due to ghost points. This may constitute an additional source of error. Finally, the co-registration accuracy of the individual scans falls within 2.3 mm. including both horizontal and vertical errors. Horizontal co-registration errors have no effect on crown height percentile calculations since the point clouds representing the individual trees were cut out with the same 2D and height boundaries each time, allowing for at least 10 cm distance around the points observed to belong to the tree. Vertical co-registration errors might have an effect, but that is well characterized by the error observed at the co-registration targets since the reference spheres and the greenhouse structure were further from the sensor than the trees were. Co-registration errors would reveal themselves by spatial autocorrelation, which we did not detect in our data. In this study we followed the methodological approach used by Puttonen et al. (2016) as close as possible to make the results comparable. The relatively low number of measurement points on the trees limited more sophisticated analysis of position change, e.g., by segmentation or the iterative closest point algorithm (Glira et al., 2015).

The phase-based TLS successfully recovered sleep movement in some of the trees species studied, whereas no movement was detected in other of the trees. The small size of the trees included in the experiment probably constrained the magnitude of the movement. The scannings were not duplicated for practical reasons (for most species, the gardening shop had only one individual in stock) except for the two individuals of *P. nigra*, thus we cannot exclude that some of the canopy movements were undetected (false negatives). Also due to the small size of the trees it was decided not to affix standard reflectors to the branches that would have allowed for a more accurate tracking of movement. The draw-back of using such a method would have been that the weight of these reflectors could have influenced the movement patterns. Although the half-hour intervals between some scans represent a step forward compared to Puttonen et al. (2016), the timing of the survey was still not optimal. The duration of the experiment should ideally be 24 h, but for the interpretability of the results the scannings should at least have been initiated a couple of hours before and run for a few hours longer. Despite the exploratory nature of this study we have nevertheless revealed substantial variation of nocturnal plant movement across seed plants, which opens up a number of new research questions specifically addressing the dynamics of sleep movements in trees.

### A conceptual framework for interpretation of plant movement

Our results clearly show that small trees move their canopy during the night in complex ways and that it is difficult to identify general patterns. Some of the movements detected were close to the preset noise threshold and although we find it unlikely, we cannot exclude effects caused by random noise. Based on the results of the present exploratory study we propose a framework that can be used for future reference of research focusing on the morphological and physiological implications of canopy movements. We propose that all movements are broken down in four basic and more or less interacting patterns, (1) sleep (circadian, light or internal clock controlled), (2) drift (non-circadian, a trend within the window of observation), (3) oscillation (short term fluctuation, typically superimposed on other types of movements) and, (4) noise (random fluctuation around a mean of zero within the expected noise limits).

As an initial rule of thumb we differentiated between canopies with and without circadian movement (“sleep”) respectively by comparing the starting and end positions of these. Traditionally sleep movement has been defined as vertical, cyclic with approximately 12 h as period length (Darwin and Darwin, 1880; Puttonen et al., 2016). We classified movement as sleep if the tree returned to its starting position 8–12 h after the start of the experiment, and if one or two clear minimum/maximum height positions could be identified. Most of the trees included in this study returned to their evening positions the morning after thereby moving full circle, whereas others showed unidirectional movement and never resumed their position at the beginning of the experiment. Sleep movement governed by an internal circadian clock has already been described e.g., in *Arabidopsis thaliana* (Barak et al., 2000; McClung, 2013). Canopy movement may be an adaptation to light exposure, and may occur in both upward or downward directions during the night. The movement pattern during the day is expected to be the opposite (Mullen et al., 2006). The movement itself is linked to turgor pressure in cells situated in places of the plant body where their effect on organ motility is optimal. This applies e.g., to the bulliform cells in grass leaves, the lodicules in grass flowers and to cells in the pulvinus found in plant families such as Fabaceae and Maranthaceae that controls movement of the leaf lamina. When it comes to movement of branches, much less is known but this is most likely a consequence of the hydraulic dynamics of the conducting tissues. The simplified view that phloem and xylem constitute separate transport systems with exclusive functions has recently been abandoned and today, we have a more refined understanding of the complex interaction between tissues of the inner bark region and the xylem, facilitated by radially aligned rays (Pfautsch et al., 2015). The palm included in this study is of course excepted since it is a monocot without rays. It has been shown that the diurnal pulse of stem expansion and contraction is driven by the daily cycle of transpiration and to a lesser degree by photosynthesis (Zweifel et al., 2001). The mechanisms behind the minute nocturnal branch movements revealed in this study are probably more complex and linked to physiological dynamics.

The second type of movement we propose here is “drift,” which implies unidirectional movement. Observed movement was classified as drift if no periodic changes in movement direction occurred and the position farthest from the starting point was measured at one of the last scans in the morning. Since we only ran our experiment for 12 h we do not know whether these movements form part of a long-term change in the tree shape. Such long-term changes may be linked to stress caused by disease, herbivory, or lack of water and will eventually lead to wilting. It may also be a result of the plants preparations for hibernation. The study was conducted near autumnal equinox to assure an almost even length of night and day and we cannot exclude that some of the trees were already preparing for hibernation.

The third type of movement, which we refer to as “oscillation” includes short-term movements in the canopy that occur in cycles of a couple of hours or shorter. Movement was interpreted as oscillation if at least three local maxima or minima could be observed, with comparable amplitudes. Although the biomechanics and ecophysiological basis of these are unknown, movements are most likely linked to tissue specific changes in turgor pressure caused by action of membrane borne ion pumps. We can only speculate of the molecular mechanism leading to these rather fast changes in turgor pressure, yet one possibility could be pulses in the way osmotically active metabolites are stored and released. In that case the movements could be regarded as *consequence* of physiologically processes that are vital to the plants and the movement *per se* can therefore be regarded as having no adaptational implications.

Lastly random noise should also be considered as a driver of movement recorded. Here, we used a preset threshold of 5 mm to filter out the noise, well knowing that we could lose valuable information in this way. For convenience and lack of knowledge we used the same filter across all the plants studied. Future studies of plant movement should consider other ways of reducing noise especially in those species that show moderate changes in the canopy.

Observed movements are a result of a combination of these four types, therefore their interpretation might still be ambiguous. Combinations of two or three main movement types may be superimposed and can in such cases only be interpreted through the main trend and local deviations from this trend with shorter or longer periodicity.

### Interpretation of our observations based on the conceptual framework

Among the species included in this studies *A. palmatum* and *A. hippocastanea* most clearly represent the sleep type of movement. In both species drift is negligible, whereas oscillations are superimposed on the general sleep pattern. The latter is most obvious in *A. hippocastanea*. Interestingly, both species are classified in the *Sapindaceae* in the malvid clade, and both have palmate, deciduous leaves. However, the leaves of *A. hippocastanea* are much larger and therefore contribute more to the movement of the crown. *V. pragense* and *O. europea* are also primarily showing sleep movement. The crown moves upwards in the early hours of the night and back down by morning. We were unable to distinguish drift or oscillations from the background noise. This was also the case for *P. x fraseri*, which nevertheless seems to have a short period of active oscillation movement before and especially after an 8-h period of sleep. In our study, *N. oleander* showed downward circadian movement, reminiscent of the movement identified by Puttonen et al. (2016) in *B. pendula*. The crown of *Albizia* shifted upwards until 8:30 p.m. and then downwards until it reached the lowest point at 03:00 a.m. After it had resumed the baseline position at 5:30 a.m., it continued upwards. This overall pattern in combination with leaf movement suggests that this species has a pronounced sleep cycle, but that the window of observation in our study was too short to completely capture it. These findings suggest that sleep movement can occur in trees both in downward direction as revealed by Puttonen et al. (2016) in *B. pendula*, as well as in the upward direction, or downward for part of the branches and upward for the rest as we observed for *Olea* and *Viburnum*. We can only speculate about the mechanisms leading to these differences and more plant physiological and biomechanical studies are needed to fully understand the driving mechanisms.

Clear cases of drift were revealed in *Morus alba* and *F. murielae*, with *Morus* moving upwards and *Fargesia* moving downwards. We cannot rule out that these plants later returned to their starting position since the experiment only lasted 12 h. The *Morus alba* tree revealed several signs of being unhealthy, which suggests that the unidirectional movement was a result of wilting of the plant. However, this was not evident in the *Fargesia murielae* and furthermore this bamboo showed signs of reversal to the starting configuration of the crown between 4:30 a.m. and 5:30 a.m. The fabaceous tree *Gleditschia* started moving downward in the evening and reached a low point at 8:00 p.m. Then it moved upward during the remaining part of the night and reached the most elevated position in the morning. This movement pattern can best be explained by downward sleep such as in *A. julibrissin*, but combined with an upward drift. Similarly the results suggest that *P. virginica* has upwards sleep movement. During early morning, the leaves started resuming the evening position. It is not clear whether they complete the full circle of movement, or whether the upward movement is repeated resulting in overall drift. In case of the palm tree *T. fortunei* the movement of the crown can be entirely attributed to the leaves. Our observations suggest an upward sleep movement for the upper part of the crown combined with a downwards drift of some of the lower part.

The sleep cycle of *B. utilis* is reminiscent of that described for *B. pendula* by Puttonen et al. (2016). It differs from this by having a barely distinguishable upwards drift superimposed.

Some trees revealed cyclic movements of shorter duration than the sleep movement, which we refer to here as oscillation. In two species we captured crown movements in 3 or 4 h cycles. This was most evident in *Magnolia alba* and somewhat obscure in *Q. robur*, being barely above the preset level set for background noise.

The gymnosperms mainly differed from angiosperms trees by the short-period fluctuations, which were more pronounced and spatially decoupled in the sense that the crown height percentiles did not show concerted movement such as it is more or less the case with angiosperms, but moved rather independently of each other. The results indicate that “sleep” movements may be weak or entirely absent in gymnosperms. The large individual of Austrian pine (*P. nigra* 1) revealed the clearest patterns of short-period movement of which individual pulses reached 0.6 cm. The small Austrian pine tree (*P. nigra* 2) had a similar pattern of erratic pulses deviating from the starting configuration at night. The individual percentiles of *C. libani* showed distinct peaks, whereas the mean of the percentiles was less clear and difficult to interpret. *L. kaempferi* was the only gymnosperm with movements reminiscent of “sleep” cycle. Superimposed on this pattern we captured an oscillation effect, which exceeded the preset background noise level for the individual percentiles as well as for the mean of all percentiles.

### Comparison with taxonomy, morphology, and anatomy

The two representatives for the malvid clade, *A. hippocastanea* and *A. palmatum* showed similar sleep patterns. Superimposed on this pattern *A. hippocastanea* showed conspicuous oscillation, which we attribute to the larger leaves of this species as compared to *A. palmatum*. *Olea* and *Viburnum* both have rigid, sclerophyllous leaves, which may explain why no short-period movement was observed in these plants. *Photinia* also seems to match this pattern of no short-period movement, at least during its sleep period, which is shorter than for other trees. The three Fabaceous trees probably have downward sleep cycles, but the duration of the experiment was probably too short so the drift observed may actually instead be part of a longer sleep cycle. This may also apply to the drift recorded in *P. virginica and B. utilis*, which may have sleep cycles lasting longer than the duration of the experiment.

Apparently gymnosperms have no sleep movement, except for the single case of the smaller *L. kaempferi* in which sleep movement cannot be cannot entirely ruled out. *L. kaempferi* stands out among the gymnosperm species investigated by being deciduous and having needles with less supportive tissue. In general we saw a gradual dimming of pulsation movement from *P. nigra* through *C. libani* to *C. nootkatensis*, which is probably correlated to decreasing length of the needles. Interestingly, *M. grandiflora* is the only angiosperm where short-term oscillation is overriding the circadian rhythm. This species represents a basal lineage within the angiosperms and as such has an isolated taxonomic position.

To sum up: sleep is variable in both direction (upward or downward) and extent and apparently determined by ancestry to a certain extent. Oscillation, which may be a universal feature, is probably controlled by the amount of supportive tissue in the leaves. Finally, drift is probably related to physiological phenomena such as, growth, senescence, and health of the plant.

### New finding and hypotheses

The demonstration by Puttonen et al. (2016) of sleep movements in two provenances of *B. pendula* left the question outstanding whether it is a widespread phenomenon across seed plants. Our results indicate that several types of circadian crown movement occur and that some trees move more than others. Some of the movement patterns detected were quite unique but at the same time so close to the preset detection threshold that we abstain from sweeping conclusions, but instead encourage further studies. The fact that our experiment only lasted 12 h may in some instances have prevented us from capturing the full extent of the sleep cycles and instead led us to interpret the observed pattern as drift since the tree did not resume the starting position at the end of observation. Puttonen et al. (2016) hypothesize that the observed branch movements are independent from sunlight and governed by the internal circadian clock of the plant. Here, we also show ample cases of canopy movement taking place during the dark period of night after sunset and before sunrise. The results of the present study suggest that movements are somehow linked to ancestry. Biomechanics and ecophysiology are probably key drivers that interplay in complex ways, but this needs to be further explored before speculations are made.

Nevertheless nocturnal crown movement is a general characteristic of the plants monitored in this study. More specifically in nearly all the plants included we observed the same crown movement cycles of a few hours duration, which were also revealed (but not discussed) by Puttonen et al. (2016). Circadian movements in plants have been explained by attributes of the diurnal rhythm of photosynthesis and the resulting fluctuation in turgor pressure (Solomon et al., 2010). Does this imply that shorter cycles of movements can be attributed to short term fluctuations in turgor pressure of yet unknown causes? Sap flow measurements suggest that fluctuations in turgor pressure more or less follow the diurnal rhythm in light exposure of the plant (Chapotin et al., 2006; Steppe et al., 2015). However, Ehrenberger et al. (2012) revealed some instances of short cycle fluctuations in turgor pressures during the night.

### Future outlook

Our experiment is a trade-off between small-sized trees grown in containers under controlled conditions and fully developed trees growing in their natural environment where larger movements are expected due both to physiological changes and to wind. Due to the extremely high point densities the datasets generated in this study allow for more sophisticated analysis and as a consequence we have made all point clouds available online for further studies. Based on our results we clearly recommend that the duration of future experiments is more than 12 h, ideally 24 h. A draw-back of the laser scanning technique that we employed here is that the point cloud is rather unstructured. At the given resolution it is not trivial to keep track of individual parts of the trees and compare these across the species studied. Puttonen et al. (2016) solve this by affixing standard measurement targets to some branches, which are then automatically located in post-processing. Alternatively, several processing techniques have been developed with the purpose of creating a 3-dimensional model from point clouds of trees obtained by TLS (Xu et al., 2007; Raumonen et al., 2013; Hackenberg et al., 2015), and these could potentially allow determining and tracking similar parts of the tree in consecutive scans to track their movement. However, such methods typically focus on modeling the branch structure without the leaves, which might be a limitation for detecting canopy movement. Alternatively, leaf segmentation approaches are also promising for detecting change maybe even at the individual leaf level (Béland et al., 2014; Koma et al., 2017). It seems feasible to alternatively use reflective prism foil for marking specific points on the branches, which can then be detected based on signal amplitude. Such a setup would furthermore open up for using a geodetic total station that can automatically locate and accurately measure the position of the reflective markers only. This would probably be a cheaper and more practical approach while delivering data that allows for similar or even more detailed spatial statistical analysis.

From a plant physiological point of view, the most daunting challenge is to explain the short-term cycles of movement. This can be done by using non-invasive methods to measure oscillations in water potential. The question is whether these are sufficiently sensitive to measure relatively small differences within a short time span. Another option is semi-invasive, measurement of conductivity between electrodes inserted into the trunk has been proposed as a metric of sap flow (Koppán et al., 2000). High precision measurements of tree girth may also give an indication of sap flow and pressure regulation but the question is again whether it is sufficiently precise enough to reveal any patterns (Pesonen et al., 2004). Finally, more invasive methods could be considered involving anatomical sectioning of fresh wood sampled at different phases of crown movement and immediately frozen in liquid nitrogen for later microanalysis of sugar contents at the tissue level.

Recent advances in laser technology open up for detailed studies of spatial dynamics in plants such as presented here. Since studies like this are still in their infancy they raise more questions that they answer. However it is very clear that plant movement is understudied, and that a set of promising methods exist that, in combination with TLS, can be expected to provide fundamental new knowledge on the physiology of plants. Our results give an impression of complex relationships that are controlled by many drivers and perhaps when mapped in detail reveal information on the plants adaptation to the environment and its interactions with other species. We have tried to reduce this complexity by establishing a rough conceptual framework within which phenomena can more easily be described.

## Author contributions

AZ planned and coordinated the study, performed data processing and analysis and wrote most of the main text. BM carried out the laser scanning measurements and the pre-processing of the point clouds, and wrote the methodology of the scanning. AB contributed to the analysis and interpretation of the results and to the discussion section. All authors worked together to produce the final version of the text.

### Conflict of interest statement

The authors declare that the research was conducted in the absence of any commercial or financial relationships that could be construed as a potential conflict of interest. The reviewer FB and handling Editor declared their shared affiliation.
