# Supplementary material for: Not All Trees Sleep the Same—High Temporal Resolution Terrestrial Laser Scanning Shows Differences in Nocturnal Plant Movement
Source: Front Plant Sci. 2017 Oct 20;8:1814. doi: 10.3389/fpls.2017.01814 (PMC5654925; doi:10.3389/fpls.2017.01814)
Supplement: Supplementary file 1 [file DataSheet1.pdf]

Supplementary Material

Not all trees sleep the same – high temporal resolution terrestrial laser scanning shows differences in nocturnal plant movement

András Zlinszky[1,3*], Bence Molnár[2], Anders S. Barfod[3]

[1]Balaton Limnological Institute, Centre for Ecological Research, Hungarian Academy of Sciences, Hungary

[2]Department of Photogrammetry and Geoinformatics, Budapest University of Technology and Economics, Hungary

[3]Ecoinformatics and Biodiversity Section, Department of Bioscience, Aarhus University, Denmark

*** Correspondence:** András Zlinszky: zlinszky.andras@okologia.mta.hu

1 Supplementary Figures and Tables

1.1 Supplementary Figures

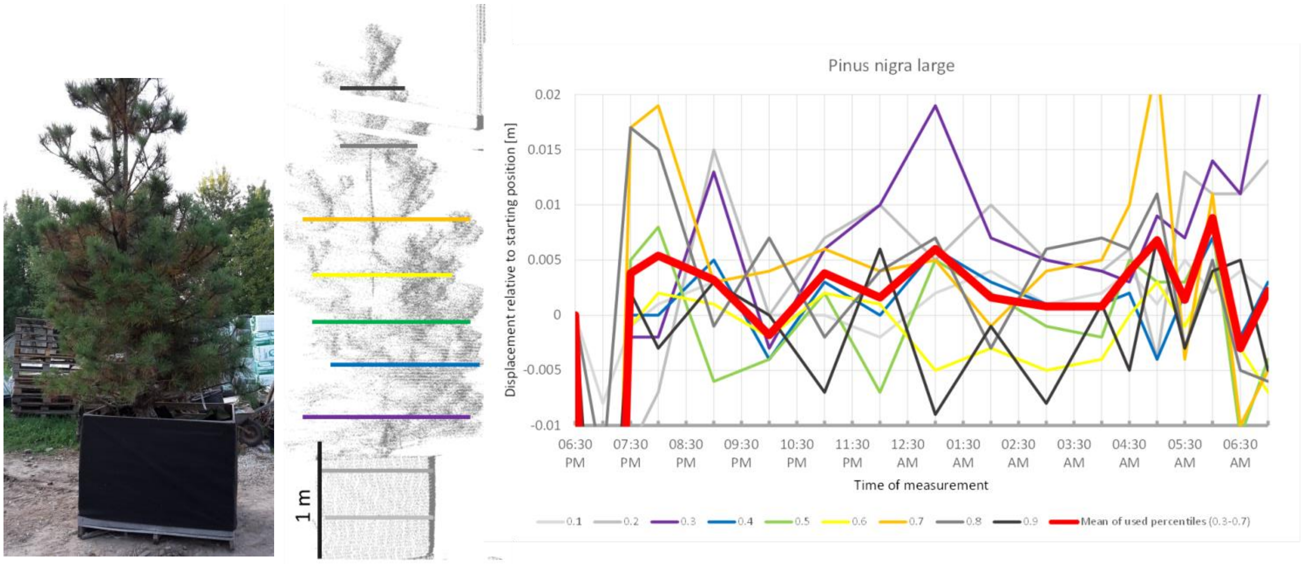

Figure S1: Point cloud height percentiles and their displacement [m] in time relative to the starting position, for the larger *Pinus nigra* tree. Percentiles not used for calculating mean movement are shown in grey. Initial extreme downward movement is an artefact caused by handling of the tree.

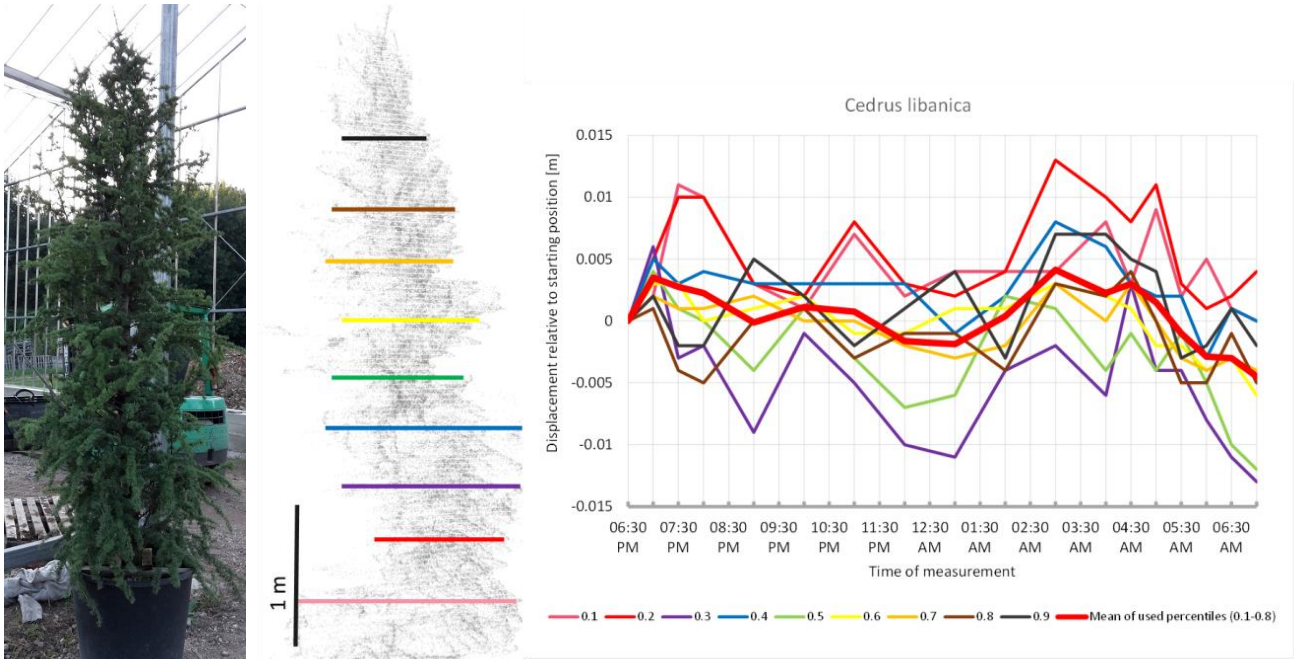

Figure S2: Point cloud height percentiles and their displacement [m] in time relative to the starting position, for *Cedrus libanica*. This tree has a near-cylindrical crown shape, so all height percentiles were used for calculating the mean displacement.

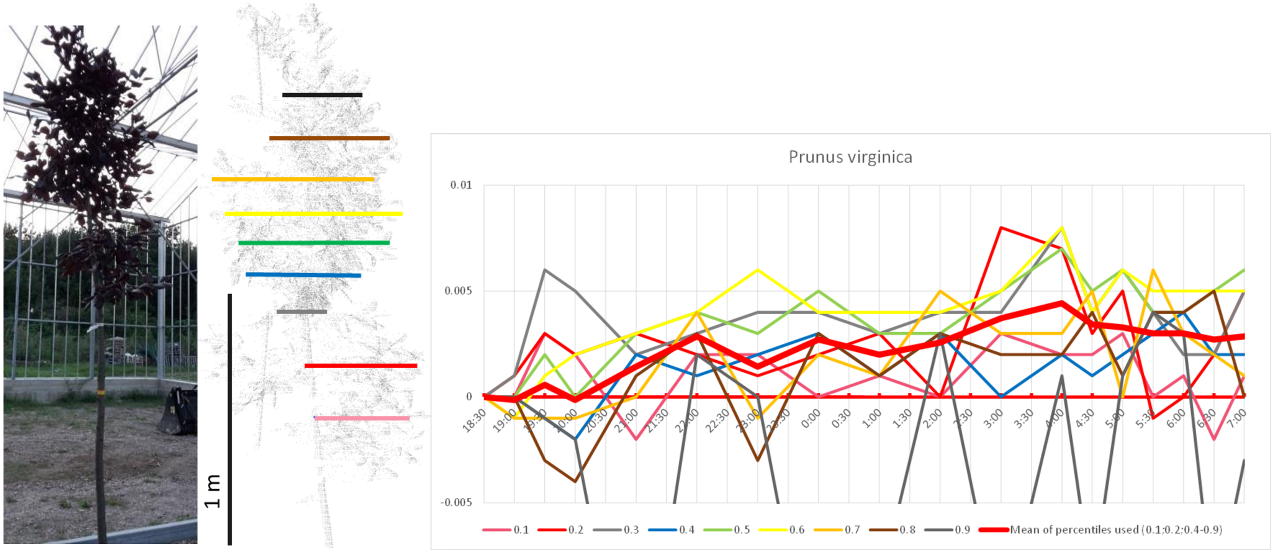

Figure S3: Point cloud height percentiles and their displacement [m] in time relative to the starting position, for *Prunus virginica*. Percentiles not used for calculating mean movement are shown in grey. Movement may be interpreted as upward sleep, but measurement period was too short to determine if the start position is reached again.

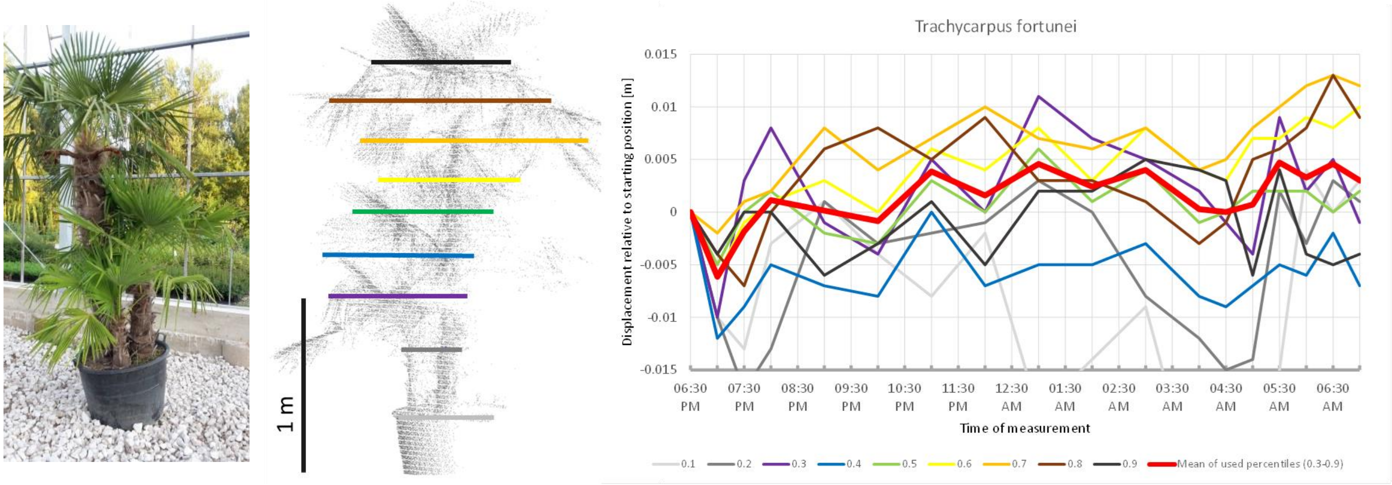

Figure S4 Point cloud height percentiles and their displacement [m] in time relative to the starting position, for *Trachycarpus fortunei*. Percentiles not used for calculating mean movement are shown in grey.

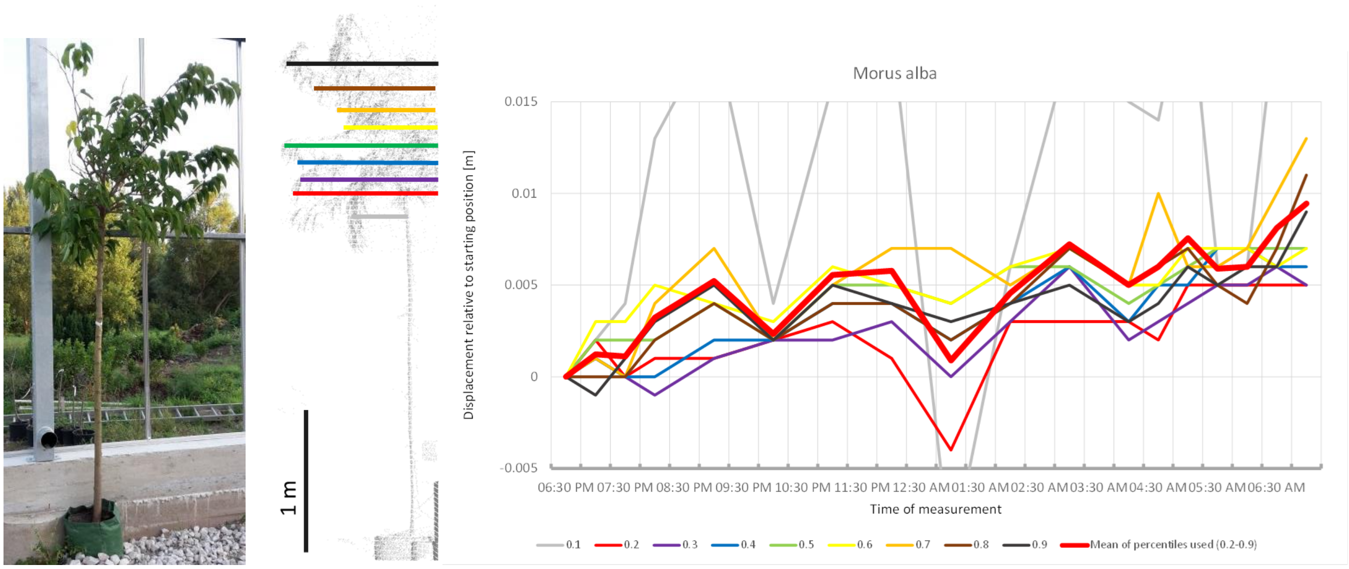

Figure S5: Point cloud height percentiles and their displacement [m] in time relative to the starting position, for *Morus alba*. Percentiles not used for calculating mean movement are shown in grey. Movement of this tree seems to be dominated by aperiodic displacement, which might be related to the wilting or senescence of this tree.

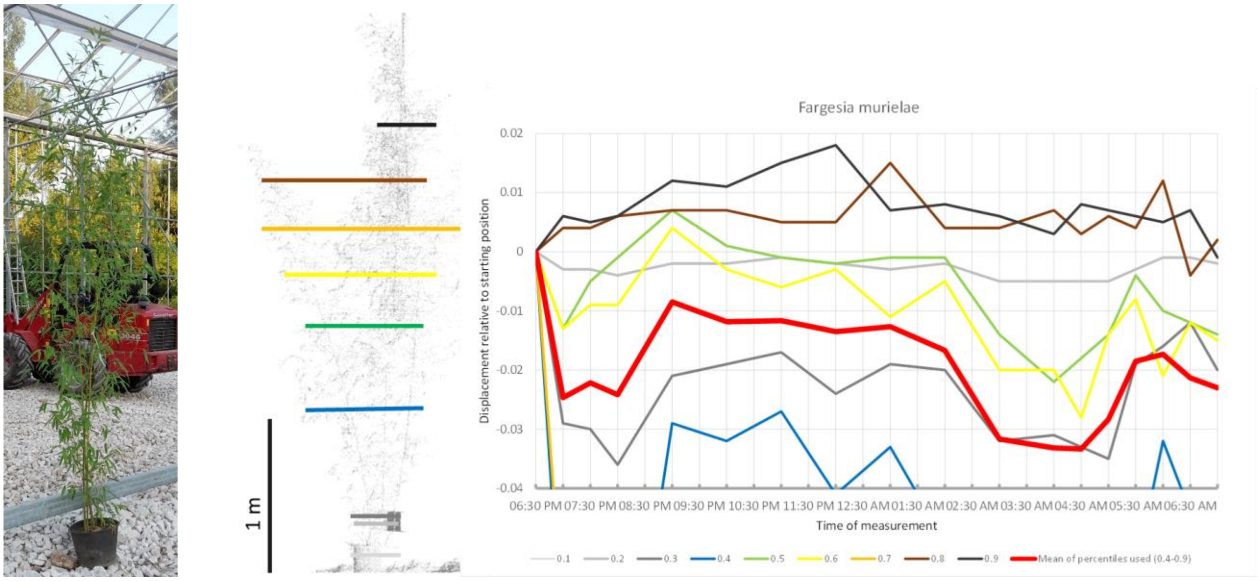

Figure S6: Point cloud height percentiles and their displacement [m] in time relative to the starting position, for *Fargesia murielae*. Percentiles not used for calculating mean movement are shown in grey. Movement of this tree seems to be dominated by aperiodic displacement. Note that upper percentiles move upward and return to start position while lower percentiles move mainly downward and do not return.

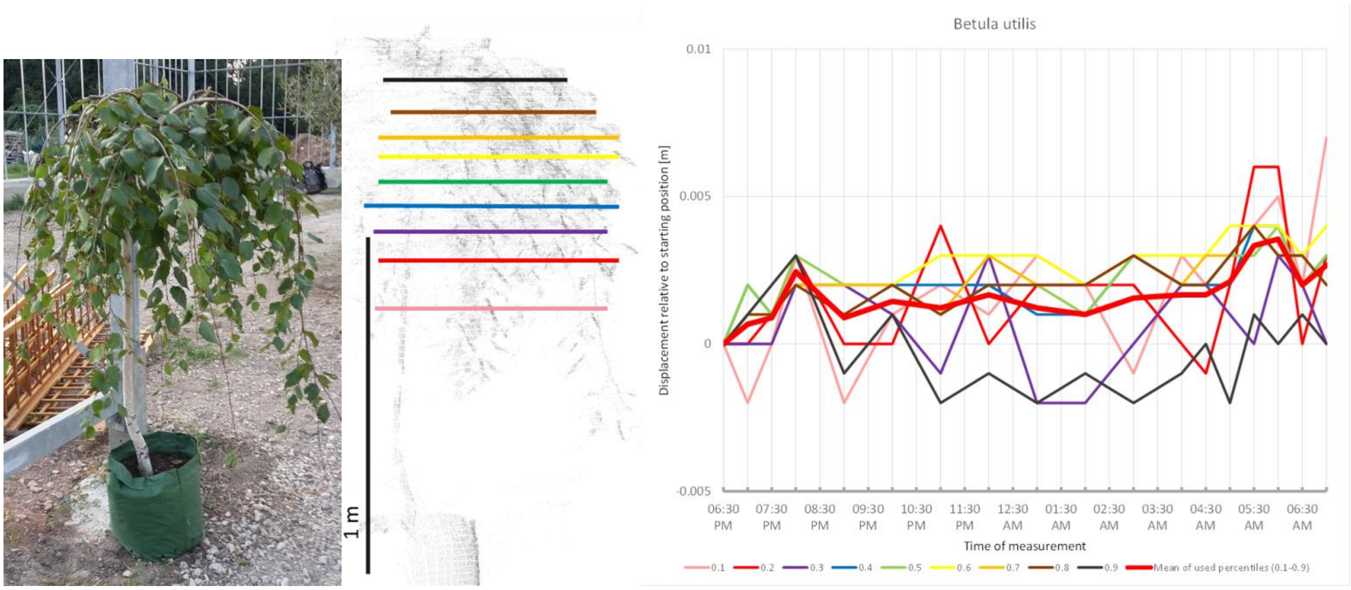

Figure S7: Point cloud height percentiles and their displacement [m] in time relative to the starting position, for *Betula utilis*. Percentiles not used for calculating mean movement are shown in grey.
